# The Transcriptional Network That Controls Growth Arrest and Macrophage Differentiation in the Human Myeloid Leukemia Cell Line THP-1

**DOI:** 10.3389/fcell.2020.00498

**Published:** 2020-07-03

**Authors:** Iveta Gažová, Lucas Lefevre, Stephen J. Bush, Sara Clohisey, Erik Arner, Michiel de Hoon, Jessica Severin, Lucas van Duin, Robin Andersson, Andreas Lengeling, David A. Hume, Kim M. Summers

**Affiliations:** ^1^The Roslin Institute, The University of Edinburgh, Edinburgh, United Kingdom; ^2^RIKEN Center for Integrative Medical Sciences, Kanagawa, Yokohama, Japan; ^3^Bioinformatics Centre, University of Copenhagen, Copenhagen, Denmark; ^4^Max Planck Society – Administrative Headquarters, Munich, Germany; ^5^Mater Research Institute – University of Queensland, Translational Research Institute, Brisbane, QLD, Australia

**Keywords:** macrophage, monocyte, THP-1 cells, differentiation, transcriptome, cell cycle, p53

## Abstract

The response of the human acute myeloid leukemia cell line THP-1 to phorbol esters has been widely studied to test candidate leukemia therapies and as a model of cell cycle arrest and monocyte-macrophage differentiation. Here we have employed Cap Analysis of Gene Expression (CAGE) to analyze a dense time course of transcriptional regulation in THP-1 cells treated with phorbol myristate acetate (PMA) over 96 h. PMA treatment greatly reduced the numbers of cells entering S phase and also blocked cells exiting G2/M. The PMA-treated cells became adherent and expression of mature macrophage-specific genes increased progressively over the duration of the time course. Within 1–2 h PMA induced known targets of tumor protein p53 (TP53), notably *CDKN1A*, followed by gradual down-regulation of cell-cycle associated genes. Also within the first 2 h, PMA induced immediate early genes including transcription factor genes encoding proteins implicated in macrophage differentiation (*EGR2, JUN, MAFB*) and down-regulated genes for transcription factors involved in immature myeloid cell proliferation (*MYB, IRF8, GFI1*). The dense time course revealed that the response to PMA was not linear and progressive. Rather, network-based clustering of the time course data highlighted a sequential cascade of transient up- and down-regulated expression of genes encoding feedback regulators, as well as transcription factors associated with macrophage differentiation and their inferred target genes. CAGE also identified known and candidate novel enhancers expressed in THP-1 cells and many novel inducible genes that currently lack functional annotation and/or had no previously known function in macrophages. The time course is available on the ZENBU platform allowing comparison to FANTOM4 and FANTOM5 data.

## Introduction

11q23 chromosomal translocations fusing the mixed lineage leukemia (*MLL*) gene (official gene symbol: *KMT2A*) with any one of >60 partners lead to aggressive leukemias with poor prognosis (Liang et al., 2017; Prange et al., 2017). One of the two most common fusion partners *AF9* (official gene symbol: *MLLT3*) is involved in the primary translocation in the widely studied acute myeloid leukemia (AML) cell line, THP-1. THP-1 cells were originally established from peripheral blood cells of a 1 year old boy with an AML-M5 leukemia carrying a t(9;11)(p22;q23) translocation (Tsuchiya et al., 1980). A subsequent study demonstrated that upon treatment with phorbol esters, THP-1 cells become adherent and acquire many functional characteristics of mature macrophages (Tsuchiya et al., 1982). Partial differentiation could also be elicited in these cells with the therapeutic agonist all-trans-retinoic acid (ATRA) (Chen and Ross, 2004). Antisense down-regulation of the *MLL-AF9* fusion transcript in THP-1 cells leads to growth inhibition but does not drive differentiation (Pession et al., 2003; Suzuki et al., 2009). On the other hand, differentiation with either phorbol myristate acetate (PMA) or ATRA was associated with down-regulation of *MLL-AF9* and growth inhibition (Pession et al., 2003; Chen and Ross, 2004).

The *KMT2A/MLL* gene encodes an epigenetic modifier, histone lysine methyl-transferase 2A (Hess, 2004). The oncogenic activity of the MLL-AF9 fusion product is mediated in part through another epigenetic modifier, DOT1L, a histone 3 lysine 9 (H3K9) methyltransferase that in turn regulates target genes in the *HOX* gene clusters and *MEIS1* (Nguyen et al., 2011). Genome-wide analysis of MLL-AF9 binding in THP-1 cells revealed a substantial overlap with enhancers bound by RUNX1, a transcription factor that regulates myeloid differentiation and is itself commonly involved in leukemogenic translocations (Prange et al., 2017). These studies identified a novel target of MLL-AF9, the transcription factor ZNF521. In mice, ZNF521 was enriched in hematopoietic stem cells (HSC) and germ line mutation impacted stem cell self-renewal. Knockdown of ZNF521 in THP-1 cells led to cell cycle arrest and partial differentiation (Garrison et al., 2017; Germano et al., 2017). Other genes that apparently contribute to dysregulated proliferation downstream of MLL-AF9 in either THP-1 cells or in mouse models include those encoding the transcription factor SALL4 (Yang et al., 2017) and the protooncogene EVI1 (Bindels et al., 2012).

Differentiation therapy involves forcing cells to cease proliferation and undergo terminal differentiation (Sachs, 1982). Such therapy with ATRA is one of the success stories in leukemia treatment but is applicable to only around 10% of AML cases (Ma et al., 2017). THP-1 cells provide a model system to investigate other potential differentiation therapy agents in aggressive AML. The process of differentiation of THP-1 cells has been studied in detail at the transcriptomic level as a model both of inhibition of leukemic proliferation and of macrophage differentiation. Differentiated THP-1 cells are commonly used as a tractable model for human monocytes (Bosshart and Heinzelmann, 2016), recently exploited in functional genomics using CRISPR-Cas9 deletion (Goetze et al., 2017; Osei Kuffour et al., 2018; Palazon-Riquelme et al., 2018).

The original THP-1 line became adherent in response to PMA within 3 h, but with progressive adaptation to tissue culture the cells became more resistant to differentiation with adherence delayed until 48 h of stimulation (Tsuchiya et al., 1982). The line is epigenetically unstable; the relative proportion of cells expressing markers such as CD4 (associated with undifferentiated cells) and undergoing differentiation in response to PMA changes with time in culture (Cassol et al., 2006). Subclones can be selected from the parent line currently available from ATCC that restore the original phenotype and either do, or do not, respond to PMA. In order to study the process of differentiation in a population in which the majority of cells respond synchronously, the FANTOM4 consortium cloned THP-1 cells obtained from ATCC by limiting dilution and chose one subclone in which >90% of cells became adherent within 48 h of addition of PMA (Suzuki et al., 2009).

Alongside microarrays, the consortium used CAP Analysis of Gene Expression (CAGE) to identify regulated promoters across a time course of differentiation. These studies identified a cohort of transcription factor genes rapidly down-regulated following PMA addition. SiRNA knockdown of a subset of these genes (*CEBPG, CEBPA, FLI1, GFI1, HOXA9, MYB* and the oncogenic *MLL-AF9* fusion transcript) produced changes in gene expression that partly mimicked the effects of PMA (Suzuki et al., 2009). A subsequent study revealed combinatorial impacts of several inducible miRNAs that also contribute to cell cycle arrest (Forrest et al., 2010).

The central conclusion of the FANTOM4 analysis (Suzuki et al., 2009) was that numerous regulated genes contribute to a complex network in which reduced expression of anti-differentiation/pro-proliferation genes is as essential as increased expression of regulators that promote differentiation. The FANTOM5 consortium extended the use of CAGE to generate a promoter-based transcriptional atlas for humans and mice (Forrest et al., 2014) and recognized that with sufficient depth of sequencing, CAGE could also detect RNAs derived from active enhancers, termed eRNAs (Andersson et al., 2014). CAGE profiling enabled analysis of enhancer profiles of human monocyte subsets (Schmidl et al., 2014) and a dense time course of the response of human monocyte-derived macrophages to lipopolysaccharide (Baillie et al., 2017). In the macrophage time course, and in several other systems studied (Arner et al., 2015) a transient pulse of eRNA from transcribed enhancers was detected prior to the detection of promoter activity of inducible genes.

One limitation of the earlier FANTOM4 study of THP-1 differentiation (Suzuki et al., 2009) was that the depth of sequencing of CAGE libraries was not as high as in subsequent studies so that transcription from enhancer loci could not be detected. In addition, the time course was insufficient to support resolution of the temporal of waves of transcriptional regulation. The FANTOM5 studies (Forrest et al., 2014) prompted us to reanalyze the time course of differentiation of THP-1 cells using CAGE with a view to further dissection of regulated promoters, enhancers and the complex transcriptional network that drives differentiation in this model.

The cellular commitment to differentiation or division usually occurs in the G1 phase of the cell cycle and differentiation of THP-1 cells is associated with cell cycle inhibition, with accumulation of cells in G1 and in G2/M (Chen and Ross, 2004; Gray et al., 2005; Gutsch et al., 2011). The FANTOM4 analysis revealed down-regulation of cell cycle related genes in response to PMA, with promoters enriched for E2F motifs (Suzuki et al., 2009). A set of 700 genes transcriptionally regulated in the human mitotic cell cycle has recently been generated based upon transcriptional network analysis of multiple datasets, including FANTOM5, and detailed curation and validation (Giotti et al., 2018). Some of these genes have more than one promoter and have other functions unrelated to the cell cycle in specific cell types. The set includes many genes associated with DNA repair and feedback regulation. Since the hallmark of leukemia is dysregulated proliferation we have examined in detail the coordinated expression of these cell cycle genes in THP-1 cells during PMA-induced growth inhibition. The objective was to further validate some of the novel cell cycle-related genes and to identify potential loss or gain of genes or gene expression within this co-expression cluster that might contribute to leukemogenesis and could assist with the development of differentiation therapies.

## Materials and Methods

### Cell Culture and RNA Extractions

THP-1 cells (high differentiation clone 5, from the FANTOM4 consortium, passage number 8 (P8), provided by Dr. Mark Barnett, The Roslin Institute, United Kingdom) were cultured in sterile-filtered (0.2 μm) RPMI1640 medium with HEPES modification (25 mM HEPES + NaHCO_3_) (R5886 Sigma-Aldrich, Gillingham, United Kingdom), 10% heat inactivated fetal bovine serum (FBS, GE Healthcare, PAA laboratories, Pasching, Austria), 1x Glutamax (35050-038, Gibco), 1 mM sodium pyruvate (11360-039, Gibco), 1x MEM non-essential amino acids solution (11140-035, Gibco), 50 μM 2-mercaptoethanol (31350-038, Gibco) and penicillin-streptomycin (16 U/ml and 16 μg/ml, Gibco) (THP-1 medium). Cells were incubated at 37°C, 5% CO_2_ and their concentration was maintained between 2 × 10^5^ – 8 × 10^5^ cells per ml by splitting in half three times a week. For the THP-1 differentiation time course, THP-1 cells were grown up at a low passage number (typically 2 or 3 passages from the P8 stock). The day before the start of the differentiation, cells were counted by hemocytometer and for each time point between 3 × 10^6^ and 5 × 10^6^ cells were pelleted and resuspended in 10 ml fresh medium. THP-1 cells were then differentiated by adding 30 ng/ml (48.6 nM) phorbol 12-myristate 13-acetate (PMA; P1585, Sigma-Aldrich) in DMSO. The cells were plated on a Sterilin bacteriological plate and at appropriate time points, the cells were lifted off by flushing with a blunt-end needle syringe, resuspended in 1 ml of RNA Bee (AMS Biosciences, Frienswood, TX, United States) and frozen at −80°C for later extraction. Medium was not changed for the duration of the experiment to avoid initiation of a serum-specific response during the time course. For each experiment, one culture was used to set up two batches of cells. The first batch was treated with PMA on the morning of the first day. Time points between 0 and 12 h were taken from this batch. The second batch from the same culture was treated with PMA in the evening of the first day (which is the 0 h time point from this batch, taken at the same time as the 12 h time point of the first batch). All cells were then incubated overnight. On the morning of the second day, the 24 h time point of the first batch and the 12 h time point of the second batch were taken, followed by time points between 12 and 24 h taken from the second batch. Batches had several overlapping time points (0, 12, 24, and 36 h) to ensure that cells were at similar state of differentiation. RNA was extracted from the RNA Bee lysate according to manufacturer’s instructions. Extracted RNA was treated with DNase I (Ambion DNase kit AM1906, Thermo Fischer Scientific) and the quantity and quality assessed using a NanoDrop spectrophotometer ND-1000 (Nanodrop technologies, Wilmington, DE, United States) and Agilent RNA ScreenTape System (Agilent Technologies, Santa Clara, CA, United States) according to manufacturers’ instructions. Only RNA samples with RIN^e^ values (Imbeaud et al., 2005) around or above 8 (out of 10) were used for CAGE sequencing.

### Cap Analysis of Gene Expression (CAGE)

Time points chosen for CAGE sequencing were hourly from 0 to 12 h, two hourly from 12 to 18 h, and then at 24, 36, 48, and 96 h. At least three replicates of each time point were included. Eight samples of 5 μg RNA each were made into one CAGE library following the protocol adapted from Takahashi et al. (2012). All the primer sequences were taken from Takahashi et al. (2012) and primers were synthesized by IDT (Coralville, IA, United States). A detailed description of the CAGE protocol and associated bioinformatic analysis is provided in Supplementary File S1. Results for 66 samples were processed using the R/Bioconductor package CAGEr (Haberle et al., 2015). All 66 samples were normalized to a power law distribution (Supplementary File S1), with expression levels presented as tags per million (TPM). The full quality assurance process for CAGE sequencing is outlined in Supplementary File S1. TSS were mapped to the human genome, version GRCh38.p9. The normalized and aggregated groups of TSS (clusters of transcription start sites; CTSS) were then annotated with gene names as described in Supplementary File S1.

### Network Analysis of Gene Expression

Patterns of gene expression during differentiation were visualized using the network analysis tool Graphia Professional version 2.0^1^. Mean expression values across replicates for CTSS with at least 10 TPM in at least one sample were uploaded into the Graphia Professional software. Sample-to-sample analysis was performed using a correlation coefficient threshold of *r* ≥ 0.95, as indicated in the Results. Two samples, both at the 4 h time point, were found to be outliers, probably due to low level of mapped reads (2.7 and 6.2 × 10^6^ reads). These were removed from subsequent analysis. Gene-to-gene analysis was performed using a correlation coefficient of *r* ≥ 0.75. Clusters of genes with similar expression patterns were determined with the Markov Clustering Algorithm (MCL) at an inflation value (determining granularity of the clusters) of 1.7. A list of genes associated with cell proliferation was obtained from Giotti et al. (2018) and a list of human transcription factors from Lambert et al. (2018). Further network analysis was performed on these subsets of genes, with correlation coefficients and inflation values as described in section “Results”. DAVID (Database for Annotation, Visualization and Integrated Discovery^2^) was used to identify functionally enriched biological themes based on gene ontology (GO) annotations, using the Functional Annotation Clustering tool (Huang et al., 2007). High annotation scores indicate significant enrichment for groups of related GO terms.

### Analysis of Transcription Factor Binding Motifs

Activities of transcription factor binding motifs were estimated using MARA (Suzuki et al., 2009) using all CTSS with at least 10 TPM in at least one sample, excluding the mitochondrial chromosome and considering conserved motifs in the region −300 to +100 bp of the midpoint of the CTSS. Motif names are based on the set of consolidated position weight matrices for human transcription factors curated for the earlier project (Suzuki et al., 2009), available at http://swissregulon.unibas.ch/sr/fantom4.

### Analysis of Putative Enhancer Expression

Candidate enhancers were identified as bidirectional TSS as described (Andersson et al., 2014) at two levels of stringency. The more stringent set (∼1000 loci) required at least two libraries with more than one CAGE tag for each CTSS, and for the enhancers an absolute directionality score of less than 0.8, and bidirectional tags in more than two samples. For the less stringent set (∼2500 loci), the criteria were >1 tag per CTSS in at least one sample, and bidirectional tags for each enhancer in at least one sample. All bidirectional TSS regions closer than 500 bp to an annotated TSS and closer than 100 bp to an exon were removed to avoid detection of promoters of protein-coding genes. The analysis was done with use of the CAGE analysis package CAGEfightR (Thodberg et al., 2019), and the ported enhancer detection scripts from Andersson et al. (2014)^3^.

### Validation of Gene Expression Levels With Quantitative Reverse Transcriptase PCR

Gene expression levels detected by CAGE were validated for representative genes using quantitative reverse transcriptase polymerase chain reaction (qRT-PCR) on the same RNA samples. cDNA was synthesized using Superscript III Reverse Transcriptase (Invitrogen) according to manufacturer’s instructions, using 500 ng of total RNA and 100 ng of random primers. The ribonuclease inhibitor used was RNasin Plus (Promega, Madison, WI, United States; 20 units per reaction) and extension at 50°C was for 60 min. The resulting cDNA was diluted 1:1 with water. Standard curves were established using four concentrations of starting RNA: 12.5, 6.25, 3.13, and 1.56 ng/μl. qRT-PCR was carried out using SYBRGreen (Roche, Mannheim, Germany) in a Roche Light Cycler 480 according to manufacturer’s instructions. Samples were pre-incubated at 95°C for 5 min (ramp rate 4.40°C/s), then amplification steps were repeated for 45 cycles. Amplification steps were 95°C for 10s (ramp rate 4.40°C/s), 60°C for 15 s (ramp rate 2.20°C/s) and 70°C for 30 s (ramp rate 4.40°C/s). Afterward, the melting curve for primers was measured by incubating at 95°C for 5s (ramp rate 4.40°C/s), 65°C for 1 min (ramp rate 2.20°C/s) and then the temperature was increased to 97°C by 0.11 C°/s. At the end, the plate was cooled for 30 s at 40°C. Calculations were performed using the Roche Light Cycler 480 software, with the Advanced Quantification setting. ΔCt was calculated with previously established values of primer efficiencies from the standard curves (calculated using the same software, by using Abs Quant/2nd Derivative Max setting). Primer sequences and efficiencies are available in Supplementary File S1.

### Analysis of the Cell Cycle in Differentiating THP-1 Cells

Propidium iodide staining was used to examine the cell cycle. 1 × 10^6^ cells were pelleted at 400 g for 5 min and resuspended in 1 ml of ice-cold 70% ethanol and then left at 4°C for at least 24 h (up to 2 months). On the day of the flow cytometry, cells were spun down at 16,000 *g* for 5 min and then washed twice with PBS. 300 μl of the Cell Cycle Staining Solution [38 mM sodium citrate, 10 μg/ml pancreatic RNase A (Signa-Aldrich), 68 μM propidium iodide (Sigma Aldrich)] was added to the pellet. Samples were incubated in the dark for 1–2 h and 300 μl of PBS was added right before flow cytometry. Samples were analyzed on a Fortessa cytometer (BD Biosciences, Franklin Lakes, NJ, United States) using a 610 nm laser. Results were analyzed using FlowJo software version 10.0.8r1 (Flow Jo LLC, Ashland, OR, United States) by gating single viable cells by excluding doublets. The final window showed the histogram of the area of propidium iodide channel, which was examined using the cell cycle option of FlowJo software by choosing the Watson (pragmatic) type of analysis (Watson et al., 1987).

## Results

### Characterization of the THP-1 Time Course of Differentiation

PMA treatment of THP-1 high differentiation clone 5 cells was carried out multiple times to harvest RNA for CAGE sequencing. To ensure that differentiation from monocyte to macrophage phenotype had occurred, changes in morphology and attachment to the culture vessel surface were examined for each replicate. Undifferentiated THP-1 monocyte-like cells grew in suspension, with most cells not attached to the bottom surface of the vessel. The THP-1 cells started to adhere around 6–8 h after addition of 30 ng/ml of PMA. They then began to develop more macrophage-like morphology, with the most dramatic difference observed at 48–96 h. Their shape changed from being round to more variable and the cytoplasm contained more phagocytic vacuoles. At each time point there were a few cells where the morphology indicated they were more macrophage-like than the majority of cells. More than 99% of the cells were adherent and had similar morphology by the end of the time course at 96 h, indicating that this culture of THP-1 clone 5 had retained its high differentiation phenotype.

**FIGURE 1:**
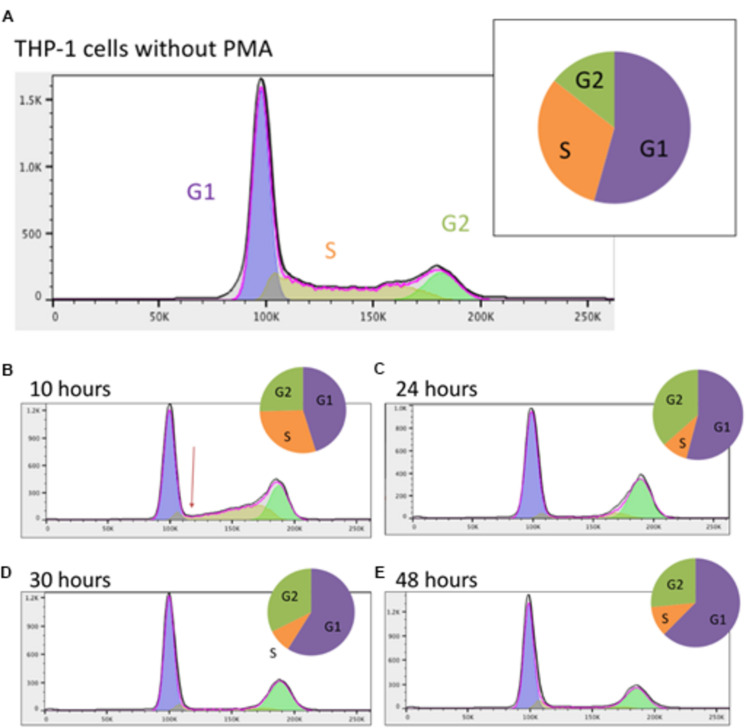
Analysis of the cell cycle in THP-1 cells. **(A)** Prior to PMA stimulation; **(B)** 10 h post PMA stimulation; **(C)** 24 h post PMA stimulation; **(D)** 30 h post PMA stimulation; **(E)** 48 h post PMA stimulation. Data were analyzed using FlowJo with the Watson (pragmatic) model. Line graph shows the number of cells in each phase, taken from FlowJo software; *X* axis shows the propidium iodide fluorescence and *Y* axis shows cell count. Arrow in **(B)** highlights the immediate rapid decline in S phase entry in response to PMA. Pie chart shows the proportion of cells in each phase. Purple – G1 phase; orange – S phase; green – G2 phase.

Prior to addition of PMA, the majority of the THP-1 cells were in G1 (growth) phase with approximately one third in S (DNA synthesis) phase (Figure 1A). After 10 h, the profile of DNA content detected by FACS was clearly changed. The number of cells in S phase was dramatically reduced and by 24 h there were <10% of cells with intermediate DNA content between 2N (G1 phase) and 4N (G2 phase) (Figures 1B,C). This low proportion was maintained for the rest of the time course (Figures 1D,E). The proportion of cells with 4N DNA content (G2) actually increased by 24 h as cells that had already entered S phase completed DNA synthesis. The proportion of cells in G2 phase gradually declined thereafter. These results indicate that differentiation of THP-1 cells to a macrophage morphology was preceded by a rapid decline in proliferation due to blocks at both entry to S phase and the well-documented G2-M phase (cytokinesis/cell division) check point, most commonly associated with DNA damage (Stark and Taylor, 2004).

To ensure that the differentiation of THP-1 cells from pro-monocytic to macrophage-like phenotype was supported by changes in gene transcription, gene expression levels derived from qPCR were assessed for two relevant markers, *MYB* which encodes a transcription factor associated with proliferating hematopoietic cells (reviewed by Zhou and Ness, 2011) and monocyte differentiation antigen gene *CD14* (Bazil et al., 1982; Wright et al., 1990). *MYB* was down-regulated during THP-1 differentiation in the earlier FANTOM4 study (Suzuki et al., 2009). In the present study, *MYB* expression was also found to decrease after PMA stimulation in all biological replicates, with the lowest level at around 2:30–3 h (Supplementary Figure S1). *CD14*, which was not detected in undifferentiated THP-1 clone 5 cells, consistent with FACS analysis of the parent THP-1 line (Bosshart and Heinzelmann, 2016), was not up-regulated until the last few points of the time course, from 24 h (Supplementary Figure S1).

### Changes in Gene Expression During Differentiation of THP-1 Cells

**FIGURE 2:**
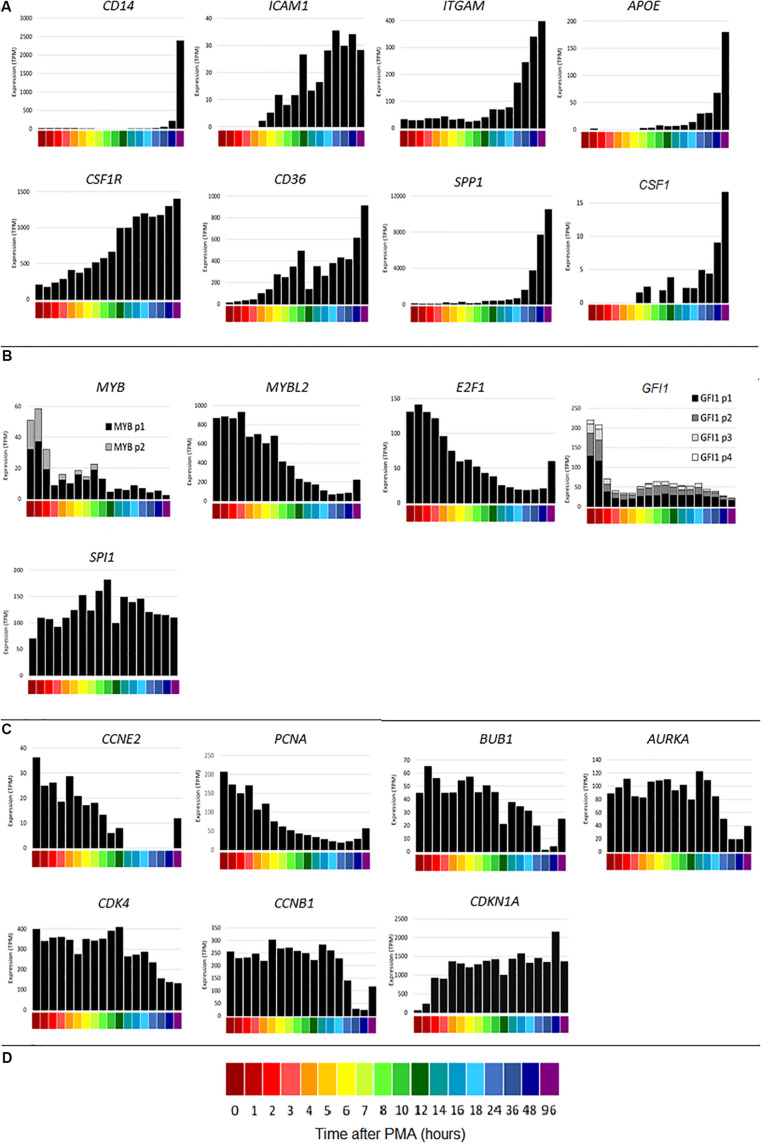
Gene expression during differentiation of THP-1 cells. *X* axis shows time points from 0 to 96 h after administration of PMA; *Y* axis shows average expression by CAGE analysis in tags per million (TPM). **(A)** Macrophage differentiation marker genes *CD14, ICAM1, ITGAM, APOE, CSF1R, CD36, SPP1, CSF1.* **(B)** Transcription factor genes *MYB* (two CTSS), *MYBL2, E2F1, GFI1* (four CTSS), *SPI1.* Multiple CTSS associated with the same gene are shown by lighter colored sections of the bars. **(C)** Inducible cell cycle repressor genes *CCNE2, PCNA, BUB1, AURKA, CDK4, CCNB1, CDKN1A.* **(D)** Color code for time points on the *X* axis.

The time course of THP-1 differentiation for all of the replicates is available on the ZENBU genome browser^4^. The browser enables gene-specific search and includes the FANTOM5 promoter and enhancer tracks and the UCSC CpG island track. To compare the detailed time course with the earlier study (Suzuki et al., 2009) that had fewer time points, CAGE sequencing results were first analyzed for index genes highlighted as markers in that study. Figure 2A shows the time courses of representative macrophage differentiation marker genes. As with the qPCR results, *CD14* increased late in the time course. Consistent with the previous data, *CD14* was most strongly induced between 48 and 96 h. *ICAM1* was induced maximally by around 24 h, *ITGAM* (encoding CD11B) increased after 12 h and *APOE* increased from around 18 h. By contrast to the earlier study, but in keeping with other results (Datta et al., 1992), the transcript encoding the growth factor receptor CSF1R was highly expressed by THP-1 cells without differentiation (200 TPM) but slowly induced a further 7-fold by PMA, reaching a maximum around 96 h. *CSF1R* is set as the default view on the ZENBU browser. The time course of *CSF1R* induction resembled the induction of *CD36*, proposed as a marker of differentiation in this system (Maess et al., 2010). *CD36* mRNA was induced by 1 h and continued to increase throughout the time course. THP-1 cells respond to CSF1R ligand, CSF1 (Datta et al., 1992) and were previously shown to express *CSF1* mRNA (Rambaldi et al., 1988). In the present study, *CSF1* mRNA was induced with a very similar time course to *CSF1R* suggesting the likely involvement of an autocrine stimulatory loop in differentiation. *CSF1* mRNA is also induced during the differentiation of primary human monocytes (Horiguchi et al., 1986) and indeed the highest level of expression in the FANTOM5 dataset is in monocyte-derived macrophages.

The transcriptional regulation of *SPP1* (encoding osteopontin), representative of a cluster of genes induced between 12 and 24 h and associated with adherence, was dissected in detail previously (Suzuki et al., 2009). *SPP1* was also massively induced by PMA in each time course replicate generated for this study. The timing of induction varied between replicates, so that the expression was highly variable between 6 and 24 h. Figure 2A shows mean data, averaged across replicates. The variation suggested that osteopontin is induced during the process of adhesion and that cluster analysis of the replicates independently might identify other genes that are involved in this key event.

A key feature of the PMA time course also dissected previously was the rapid down-regulation of genes for transcription factors involved in cell cycle regulation, notably *MYB* and *E2F1*, associated with growth arrest and induction of differentiation. Knockdown of some of these factors was sufficient to induce growth arrest and partial differentiation (Suzuki et al., 2009). Figure 2B shows a subset of these transcription factors. CAGE sequencing showed that *MYB* was expressed from two promoters, both of which were down-regulated rapidly in response to PMA. The related transcription factor gene, *MYBL2* was highly expressed at the start of the time course (16 fold higher than the combined expression of the two *MYB* promoters) and declined later than *MYB*. *MYBL2* was not assessed in the previous analysis. *E2F1* was maximally repressed by around 12 h. *GFI1* has been identified as a transcriptional repressor of target genes of the macrophage transcription factor, PU.1 (encoded by *SPI1*) (Wilson et al., 2010; Barth et al., 2019). In THP-1 cells, *GFI1* was expressed from 4 separate promoters and rapidly down-regulated by PMA (Figure 2B). Consistent with repression of entry into the cell division cycle, the G1/S cyclin gene *CCNE2* was rapidly repressed to undetectable levels (Figure 2C). Cell cycle-associated transcripts for genes associated with S phase and mitosis (Giotti et al., 2018) such as *PCNA, BUB1, AURKA, CDK4* and the M phase cyclin, *CCNB1*, declined more slowly and progressively (Figure 2C), presumably as individual cells competed the cycle (Figure 1). Many of these transcripts showed some evidence of re-expression at 48–96 h (for example *E2F1*, *CCNE2* and *PCNA* in Figure 2), which may reflect expansion of the small subset of cells that is resistant to PMA-induced differentiation even in this cloned line.

The time course of the loss of positive cell cycle regulators in response to PMA suggests that this may be a consequence rather than a cause of the block to S phase entry (Figure 1). The FANTOM 4 project did not analyze the expression of inducible cell cycle repressors. Others reported that the cell cycle inhibitor *CDKN1A* (p21WAF) was induced by PMA at the mRNA and protein level in THP-1 cells (Akashi et al., 1999; Traore et al., 2005). The induction of CDKN1A was associated with the loss of phosphorylated CDK2 protein, which was linked in turn to selective inhibition of phosphorylation of RB (retinoblastoma protein). Figure 2C shows that *CDKN1A* was induced from almost undetectable levels within 1 h and thereafter sustained at high levels throughout the time course.

These initial studies indicate that the current dataset broadly reproduces the temporal cascade of cell cycle arrest and monocyte-macrophage differentiation seen previously (Suzuki et al., 2009) and show that the increased temporal resolution of the present study was informative. We therefore undertook a refined network analysis of the data.

### Patterns of Gene Expression Throughout Differentiation

**FIGURE 3:**
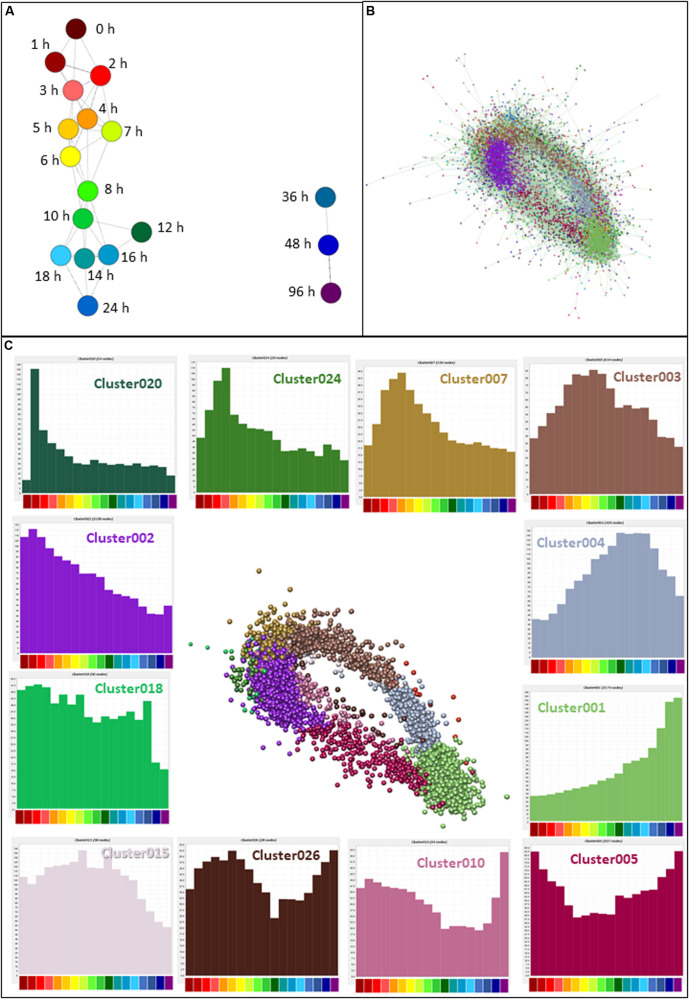
Network analysis of patterns of gene expression throughout differentiation of THP-1 cells. **(A)** Sample-to-sample analysis. Each node (sphere) represents the average of replicates at a time point, colored as shown in Figure 2D, indicating hours after addition of PMA. Edges between nodes show correlations of ≥0.95. **(B)** Gene-to-gene analysis showing the main element of the full network. Nodes represent genes (TSS) and the edges between them represent correlations of ≥0.85. MCL clustering was performed at an inflation value of 1.7 and nodes are colored according to their membership in a cluster. **(C)** Clusters of nodes that are differentially expressed across the time course. Orientation is the same as **(B)** but edges and other clusters have been removed for ease of visualization. Color of nodes shows the cluster to which nodes were allocated; histograms (colored the same as the nodes in the cluster) show the average expression patterns of the genes within the clusters. Color code for the *X* axis is as for Figure 2.

CAGE sequencing data values (means for each replicate; *N* = 2–6) were loaded into the network analysis tool Graphia Professional to find patterns of gene activation and repression across the time course. To look for general trends across all replicates, a sample-to-sample network at 18 different time points was generated using a correlation coefficient of ≥0.95. The sample-to-sample network (Figure 3A) highlights the global transcriptomic transition as the time after treatment increased. The transcriptome of samples at 0 h (deep red) were most different from those at 96 h (deep purple). Samples from other time points lie between these two, moving from red (1 h) through orange and yellow (2–6 h), green (7–12 h), and blue (14–48 h).

Next, CTSSs were correlated with each other based on the mean expression at 18 time points (to create a gene-to-gene network). The analysis was done with a correlation coefficient threshold of 0.85, which included 10,168 nodes out of 11,360 possible CTSS (see section “Materials and Methods”). Expression patterns of excluded CTSSs were not correlated with any other CTSS at a correlation coefficient of 0.85. A network graph comprising one large element of 10,046 nodes and 53 smaller unconnected elements of between 2 and 11 nodes was generated using Graphia Professional. Markov clustering with an inflation value of 1.7 partitioned the graph into clusters of CTSS with related expression profiles. Figure 3B shows the result for the main element. There were 92 clusters with 10 or more nodes. A number of clusters showed distinct patterns of gene expression during differentiation. The largest of these are shown in Figure 3C. The clusters in which the key genes discussed above were found are shown in Table 1. A full list of all the clusters and the mean expression patterns of those with 10 or more nodes is presented in Supplementary Table S1.

**TABLE 1:** Clusters associated with key genes from network analysis.

CTSS ID	Cluster number
CD14	001
ICAM1	001
ITGAM	001
APOE	001
CSF1R	001
CD36	001
SPP1	001
MYB p2	002
MYBL2	002
E2F1	002
GFI1 p1	002
GFI1 p2	002
GFI1 p3	002
GFI1 p4	002
CCNE2	002
PCNA	002
BUB1	002
CDK4	006
CDKN1A	012
SPI1	014
AURKA	273
CCNB1	273
MYB p1	332

*The analysis was based on averaged values over the whole dataset. Where present, p1 – pn indicate the detection of additional CTSS attributed to the same gene by the CAGE analysis.*

The largest cluster, Cluster001, contained genes which code for proteins associated with mature macrophage functions, including surface markers (*CD14, CD163, ITGAM*), lysosome activity, transmembrane location and integrin complex. The most significantly enriched GO term was *lysosome* (enrichment score 14.8). This cluster contained genes with expression that steadily increased throughout differentiation, peaking at the end of the time course. Cluster002 declined progressively across the time course and showed high enrichment scores for terms associated with *cell cycle* (enrichment score 31.7) and *mitochondria* (enrichment score 21.1). Three hundred and sixty seven (17%) of the genes in this cluster were contained in an annotated list of 701 cell cycle genes (Giotti et al., 2018). These two clusters are consistent with the major phenotypic impacts of PMA, growth arrest and macrophage differentiation.

Aside from these two clusters, the network analysis revealed that many transcripts were induced or repressed transiently. By 1 h after PMA treatment, early response genes with functions such as transcriptional activation were highly expressed (Cluster020 peaking at 2 h then declining rapidly and Cluster007 peaking at 4 h). Both these clusters were associated with GO terms relating to regulation of transcription (enrichment scores 2.7 and 3.9, respectively). Expression of the genes in Cluster003 began to rise after 1 h and peaked between 5 and 8 h. They were associated with both the cell cycle (enrichment score 4.9) and cell-cell adhesion (enrichment score 4.1). Expression of genes in Cluster004 slowly increased from the 2 h time point, peaking between 10 and 18 h, and this cluster was enriched for GO terms relating to lysosome (enrichment score 5.3). Images of the average expression profiles are shown in Figure 3C.

### Cell Cycle Gene Expression Throughout Differentiation

**FIGURE 4:**
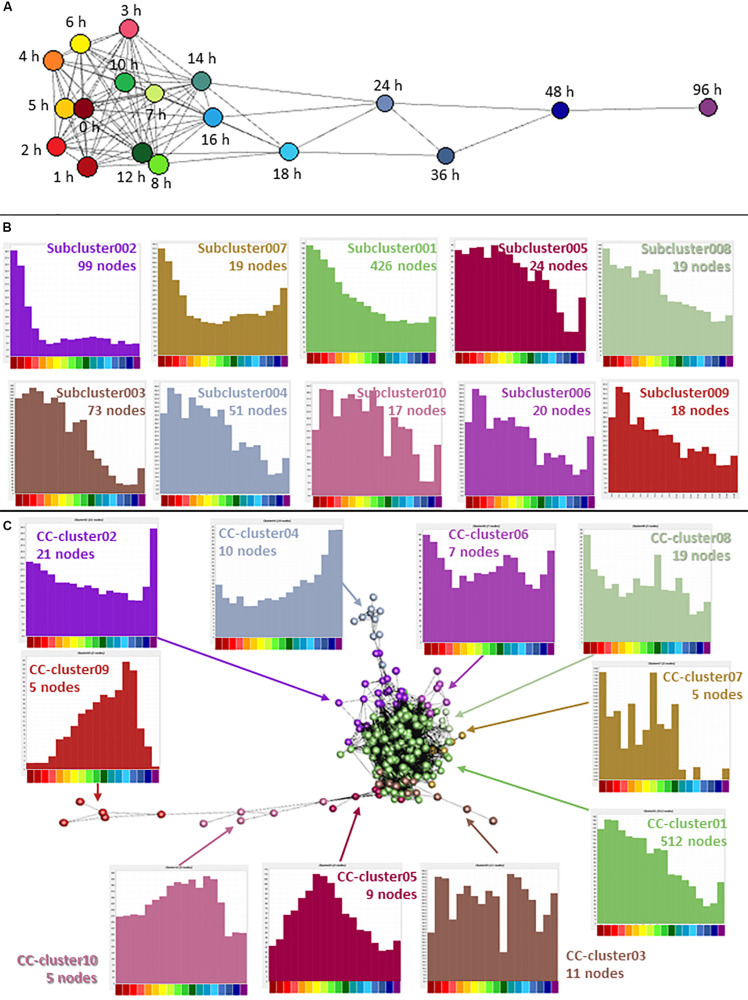
Network analysis of cell cycle genes. **(A)** Sample-to-sample analysis of Cluster002, containing genes associated with the cell cycle and proliferation. Nodes show averaged values for each time point and edges show correlations of ≥0.90 between nodes. Hours after addition of PMA are indicated. **(B)** Expression profiles of subclusters generated by gene-to-gene analysis of Cluster002. Averaged values for each time point. Correlation coefficient was ≥0.95 and MCL inflation value was 1.7. **(C)** Gene-to-gene analysis of 561 curated cell cycle genes (617 TSS) (Giotti et al., 2018) expressed during THP-1 differentiation. Nodes show averaged values for each gene and edges show correlations of ≥0.75 between them. MCL inflation value was 1.7. Histograms show the averaged expression profile for each cluster. Color code for the *X* axis is as for Figure 2.

Cancer is characterized by abnormalities in cell proliferation, particularly the escape of the cancer cells from the normal controls on cell division (Sachs, 1982). In the analysis of the whole dataset, the second largest cluster (Cluster002) contained many genes associated with cell proliferation and the averaged expression profile of this cluster showed a decline during the time course with a small increase at 96 h. Note that by contrast to earlier studies (Nguyen et al., 2011; Garrison et al., 2017; Germano et al., 2017; Prange et al., 2017) (see section “Introduction”) the set of down-regulated transcripts did not include *KMT2A* (*MLL*), *DOT1L, RUNX1* or *ZNF521*, implicated in primary leukemogenic transformation in AML. Each was expressed constitutively by THP-1 cells and unchanged upon differentiation. The cell cycle-associated cluster was explored in more detail by further clustering the expression profiles of the 2,158 genes separately, at higher stringency. A sample-to-sample analysis (*r* ≥ 0.9) showed that for this group of genes, the later time points were quite distinct, but the earlier time points (from 0 to 14 h) were relatively similar with a weak correlation between time point and position in the network (Figure 4A). The gene-to-gene analysis was run at a correlation coefficient threshold of 0.95, which included 1540 nodes connected by 17,446 edges. The network was then clustered with an MCL inflation value of 1.7. 618 nodes of the original Cluster002 were excluded at this threshold. We hypothesized that genes involved in different phases of the cell division cycle, or with different mRNA stability, might decay with distinct temporal profiles Consistent with that view, the annotated cell cycle genes appeared in specific subclusters with distinct profiles of decay with time (Figure 4B). For example, Subcluster002 with 99 nodes had none of the reported cell cycle genes (Giotti et al., 2018), a highly significant departure from expected (*P* = 7 × 10^–6^). Overall expression in this cluster dropped very early in the time course. This cluster contains monocyte-associated transcription factors (*IRF8, GFI1*). These factors, as well as *MYB* and *MYBL2* (which are also in the larger Cluster002 but did not form a part of a subcluster) have myeloid-specific roles in proliferation and down-regulation using siRNA can drive growth inhibition (Suzuki et al., 2009). Subcluster003 and Subcluster004 had a significant excess of cell cycle genes (*P* < 1 × 10^–5^ and *P* = 3 × 10^–5^ respectively). For these clusters, average expression declined slowly after 10 h and both had a small rise at 96 h, not seen in Subcluster002. Both these clusters had equal numbers of genes associated with S and G2/M phase of the cell cycle. In contrast Subcluster010 with 17 nodes had 10 G2M genes and no S phase genes (*P* = 5 × 10^–6^). Average expression was relatively flat until 18 h when it dropped rapidly. Using the Bonferroni method to correct for multiple testing, a *P*-value of 0.002 would be equivalent to the accepted threshold for significance of 0.05, so these differences are highly significant. The full list of subclusters and averaged expression profiles are provided in Supplementary Table S2.

A previous study (Giotti et al., 2018) identified 701 genes that were expressed/induced in multiple cell types specifically in S phase and G2/M phases of the cell cycle. Of these, 561 (80%) were expressed in THP-1 cells, represented by 617 promoters. These 617 promoters were selected from the filtered dataset of mean values and analyzed separately. When clustered at *R* ≥ 0.75, 599 promoters were included in the analysis and 512 of these fell into the largest cluster (named CC-cluster01) with an average expression pattern which declined slowly until 10 h and then more steeply to 48 h, after which there was a small increase in average expression (Figure 4C). This group included the classic proliferation markers mentioned earlier. This finding provides further support for the annotation of these genes as cell cycle-related. A second cluster of 21 nodes (CC-cluster02) showed a small decline in expression from 0 h, with a large increase at 96 h. Other smaller clusters containing 2 – 11 unique genes showed similar expression patterns to the whole dataset (Figure 4C). The full CC-cluster lists and averaged expression profiles are provided in Supplementary Table S3. The set of 150 cell-cycle genes for which promoter activity was not detected in THP-1 cells is provided in Supplementary Table S4. In most cases, the apparent lack of expression is an issue of promoter annotation, and there is, in fact, expression of promoter activity in the region of the corresponding gene evident in the FANTOM5 dataset. For example, our data, and the FANTOM5 data, reveal that the transcript encoding the key regulator CDC25B, which is essential for G2/M cell cycle progression in AML (Didier et al., 2008) is expressed in THP-1 cells but is primarily driven by a cluster of three promoters that are internal to the annotated TSS in RefSeqs NM_001287516, NM_001287517, and NM_001287518. This can be seen in the data available on the ZENBU browser.

In summary the analysis of known cell cycle genes is consistent with the shut-down of entry into S phase prior to the 10 h time point as indicated by the cell cycle analysis (Figure 1) and subsequent slow decay of S phase-associated transcripts as cells complete the DNA synthesis phase of the cell cycle and are blocked in G2/M.

### Transcription Factor Gene Expression Throughout Differentiation

**FIGURE 5:**
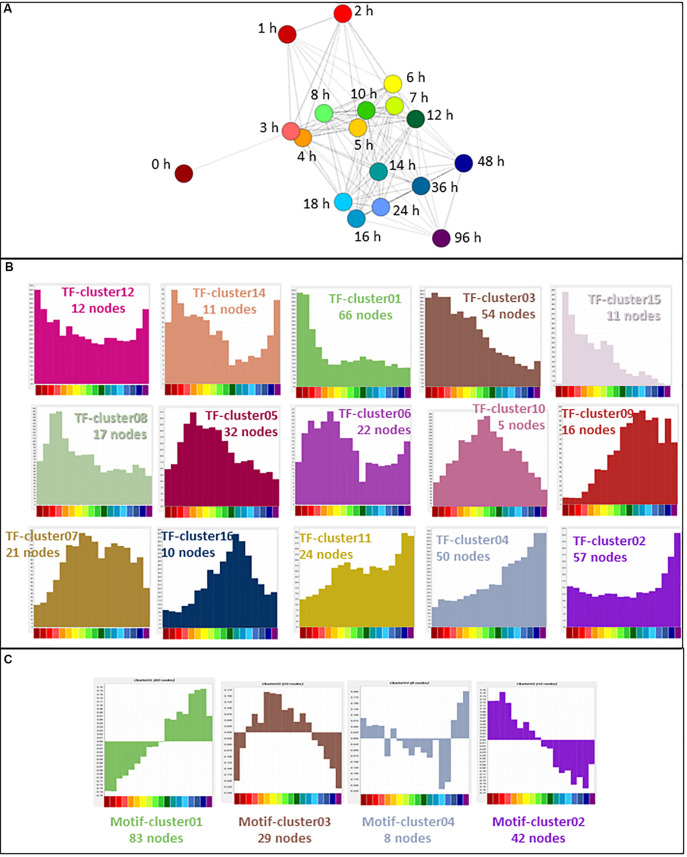
Expression of transcription factors during THP-1 differentiation. **(A)** Sample-to-sample analysis of 647 curated transcription factors (796 TSS) (Lambert et al., 2018) expressed during THP-1 differentiation. Edges show correlations of ≥0.61. Hours after addition of PMA are indicated. **(B)** Expression profiles of clusters generated by gene-to-gene analysis of curated transcription factors. Averaged values for each time point. Correlation coefficient was of ≥0.85 and MCL inflation value was 1.7. **(C)** Network analysis of transcription factor motif activity. Histograms showing average activity (as detected by MARA) of transcription factor motifs, clustered at a correlation coefficient of ≥0.60 and MCL inflation value of 1.7. Color code for the *X* axis is as for Figure 2.

In cells undergoing transitions in state, transcription factor gene expression precedes the expression of the target genes being regulated (Arner et al., 2015). Of 1639 transcription factors curated recently (Lambert et al., 2018), 647 (39%) were expressed in the filtered THP-1 dataset. The expression profiles of these genes were used for further analysis. In a sample-to-sample analysis including all time points (*R* ≥ 0.61), the 0 h time point was an outlier, linking only with the 3 h time point (Figure 5A). A gene-to-gene analysis of the 647 transcription factor genes (796 promoters) was performed at a relatively stringent correlation coefficient of *R* ≥ 0.85, which included 540 nodes (transcription factor genes) and 3026 edges (connections between them at *R* ≥ 0.85). Clustering was performed at an inflation value of 1.7 and clusters were named as TF-cluster01 etc. The clusters are shown in Supplementary Table S5 which also contains histograms of mean activity of each cluster. The much denser time course analyzed herein, compared to the FANTOM4 project (Suzuki et al., 2009) highlights a temporal cascade in which the large majority of the transcription factor promoters exhibit regulated expression. Broadly speaking, there is a set of clusters where the transcripts are progressively down-regulated with different dynamics, a reciprocal set progressively up-regulated and a third set in which expression is regulated transiently (Figure 5B).

The transiently regulated transcription factors include the so-called immediate early genes (IEG) that have been analyzed in detail in other differentiation/activation time courses by the FANTOM 5 consortium (Vacca et al., 2018). That analysis identified a set of 42 protein-coding genes with promoters that consistently drive a rapid, but transient, wave of transcription in response to stimulus. A subset of these IEG was expressed by differentiating THP-1 cells. TF-Cluster15 contains transcription factors *EGR1, EGR3, EGR4, JUNB, KLF2, KLF10* and *MEF2D* which were fully induced within 1 h and thereafter declined slowly. Other IEG had distinct patterns. *EGR2*, which has specific functions in myeloid differentiation including regulation of *CSF1R* transcription (Laslo et al., 2006; Krysinska et al., 2007), is part of TF-cluster10 (also including *ELK3, FOXP2, JUNB, TBX3*, and *ZEB2*) that peaks later around 8–10 h, around the time of the first appearance of adherent cells. *ZEB2* was shown recently to be required for the establishment and maintenance of tissue macrophage identity (Scott et al., 2018). By contrast to other systems in which it is an IEG, and consistent with its known functions in macrophage differentiation (Shen et al., 2016), *JUN* was not induced rapidly but instead formed part of a progressive differentiation cluster (see below).

Mean expression of transcription factor genes in the largest cluster (TF-Cluster01) was maximal in untreated THP-1 cells and declined rapidly in response to PMA. This down-regulated cluster contains multiple transcripts associated with immature myeloid cells and maintenance of the proliferative state, including *MYB, GFI1* (both shown in Figure 2B), *IRF8* and *RXRA.* Average expression of transcripts within TF-Cluster03 declined more slowly; they include the core regulators of cell cycle gene expression, *E2F1* (Figure 2B) and *FOXM1* (Giotti et al., 2018). Several TF clusters show progressive up-regulation with distinct profiles. TF-Cluster04 (including *JUN, MAF, NR1H3*) increased across the whole time course, TF-Cluster11 (including *ATF3, TCF7L2*) was induced earlier, and TF-Cluster02 (including *IRF1, FOXP1*) was induced late, associated with terminal differentiation. One highly induced transcription factor with a unique expression profile that did not form part of a cluster was *MAFB*, discussed further below.

### MARA Analysis

Promoters contain motifs that are recognized by DNA binding proteins. Where promoters share such motifs it is likely that they are regulated by the same proteins, and expression correlates with the number of copies of the relevant motif in the promoter region. The analysis tool MARA (Suzuki et al., 2009), which calculates motif activity based on this correlation, was used to analyze the response of THP-1 cells to PMA. The results are shown in Supplementary Table S6 and histograms of activity of selected motifs across the time course are shown in Supplementary Figure S2. A preliminary clustering analysis (*r* ≥ 0.6, MCL inflation 1.7) showed four patterns of activity: declining or increasing throughout the time course, transiently up-regulated and transiently down-regulated (Figure 5C). These patterns confirm the conclusions of the earlier work (Suzuki et al., 2009). The imputed transcriptional activity of MYB and E2F1.5 motifs, and of TFDP1, which binds cooperatively with E2F (Rubin et al., 2005), was down-regulated progressively over the time course. The activity of related motifs recognized by AP1 family members (FOS; FOS{B,L1}_JUN {B,D}; FOSL2; JUN) increased rapidly, as did the activity of the NFE2L2 and TGIF1 motifs (both factors induced by PMA).

There was some evidence of increased activity of the IRF1,2,7 motif, consistent with the induction of interferon target genes in Cluster032. Kubosaki et al. (2010) reported that the same genes were expressed constitutively in THP-1 cells and were targets of IRF8. Consistent with this and other studies (Phanstiel et al., 2017), *IRF8* was highly expressed by THP-1 cells (the most abundant transcription factor mRNA) and repressed by PMA, but the putative target genes were low in undifferentiated cells and strongly induced. PMA also initiated clear and transient increased activity of the EGR1.3 motif. Also consistent with FANTOM4 data there was a decrease in activity of the NFY{A,B,C} and octamer (POU2F1.3) motifs, although *NFYB* and *NFYC* mRNAs (both highly expressed in THP-1 cells) were not regulated by PMA.

The greater resolution of the current dataset permitted the detection of regulated activity of additional motifs, notably the NR4A2 (also recognized by NR4A1) and TFEB motifs. Both *NR4A1* and *NR4A2* were amongst immediate early genes induced by PMA. The TFEB motif, recognized by members of the microphthalmia transcription factor family, is enriched in the promoters of lysosome-associated genes (Sardiello et al., 2009; Hume et al., 2010). Two members of this family, *TFEC* and *TFE3*, were constitutively expressed in THP-1 cells but two others, *TFEB* and *MITF*, were progressively induced in response to PMA in parallel with lysosomal hydrolases (e.g., *ACP2, CTSB, LIPA*) and components of the vacuolar ATPase (e.g., *ATP6V0A1*). Conversely, we did not reproduce evidence of inducible activity of SPI1 (PU.1), RUNX1.3 or SNAI1.3 motifs although transcripts encoding the related transcription factors *TWIST1* and *SNAI1* were induced by PMA.

### Enhancer Activation During THP-1 Differentiation

**FIGURE 6:**
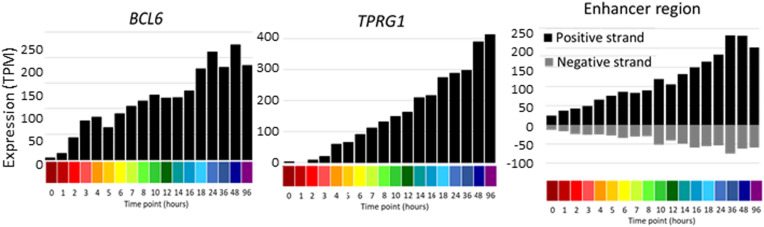
Promoter and enhancer activity of *BCL6* and *TPRG1. Y* axis shows expression levels in TPM; negative value (gray bars) indicates expression from the reverse strand. *X* axis shows the time points (hours after administration of PMA; color code as shown in Figure 2).

The increased depth of sequencing available in this dataset also enables for the first time the detection of enhancer RNA (eRNA), which derives from the transcriptional activity of regulated enhancers, and inferred relationships with inducible promoters. The eRNAs are rapidly degraded by the exosome complex (Andersson et al., 2014) and accordingly, they are generally detected at <1 TPM. Detection is therefore subject to stochastic noise with median mapped reads of around 10 million per library. Nevertheless, integration of the entire dataset enabled detection of regulated bidirectional transcription associated with enhancers annotated in the FANTOM5 dataset and identified additional candidate enhancers. The identified enhancers were compared to those identified in FANTOM 5. 1353 of the less stringent set and 656 of the more stringent set were found in the full set of ∼63,000 enhancers (Andersson et al., 2014), indicating that this analysis has discovered some potential novel enhancers. Both sets were compared to the set of ATAC-seq peaks identified in analysis of chromatin architecture of differentiation (Phanstiel et al., 2017). Overlap was detected for 88 and 79% enhancers in the more and less stringent sets, respectively. One novel example highlighted in the chromatin analysis is the complex *BCL6/TPRG1* locus. BCL6 is implicated in cell cycle regulation and interacts with TP53 and CDKN1A (Phan et al., 2005), whereas there is little known of the biology of TPRG1. The promoters of these two transcripts are separated by >200 kb (see ZENBU Browser). The TSS of *TPRG1* is located upstream of the annotated RefSeq transcript NM_198485 at Chr3:188947237, downstream of a TATA box. The differentiation time course data confirm the rapid (within 1 h) and synchronous continued induction of both transcripts, and the detectable induction of enhancers with the intervening LPP locus. Figure 6 shows the profile of the activity of each of these promoters/enhancers across the time course. A similar co-regulated pattern was evident at the *MITF* locus. The induction of *MITF* with time in response to PMA was correlated with induction of the adjacent *FRMD4B* gene and evidence of bidirectional transcription initiation in the intervening genomic region.

To further confirm the validity of the enhancers found, they were compared to every individual FANTOM5 enhancer set. The THP-1 FANTOM5 library was the top hit for both sets, and other myeloid libraries were overrepresented in the top of the list. 219 out of 1172 FANTOM5 THP-1 enhancers were confirmed in the more stringent time course set. The results are available in Supplementary Tables S7A,B.

The macrophage-specific transcription factor, PU.1 (encoded by *SPI1*) is required for differentiation and controls the expression of many macrophage-enriched genes in THP-1 cells (Suzuki et al., 2009). *SPI1* was expressed constitutively by THP-1 cells and was not highly regulated by PMA. As noted above, MARA did not reveal regulated activity of the PU.1/SPI1 motif across the time course. The expression of *SPI1* in AML cells depends upon an enhancer around 16 kb upstream of the TSS that binds the transcription factor SATB1 (Steidl et al., 2007). The CAGE data (see ZENBU Browser) reveal that there is abundant unidirectional transcription initiation from this enhancer in THP-1 cells, likely driving an annotated lncRNA, that is marginally up-regulated by PMA. *SATB1* mRNA, on the other hand, was progressively down-regulated in response to PMA.

**FIGURE 7:**
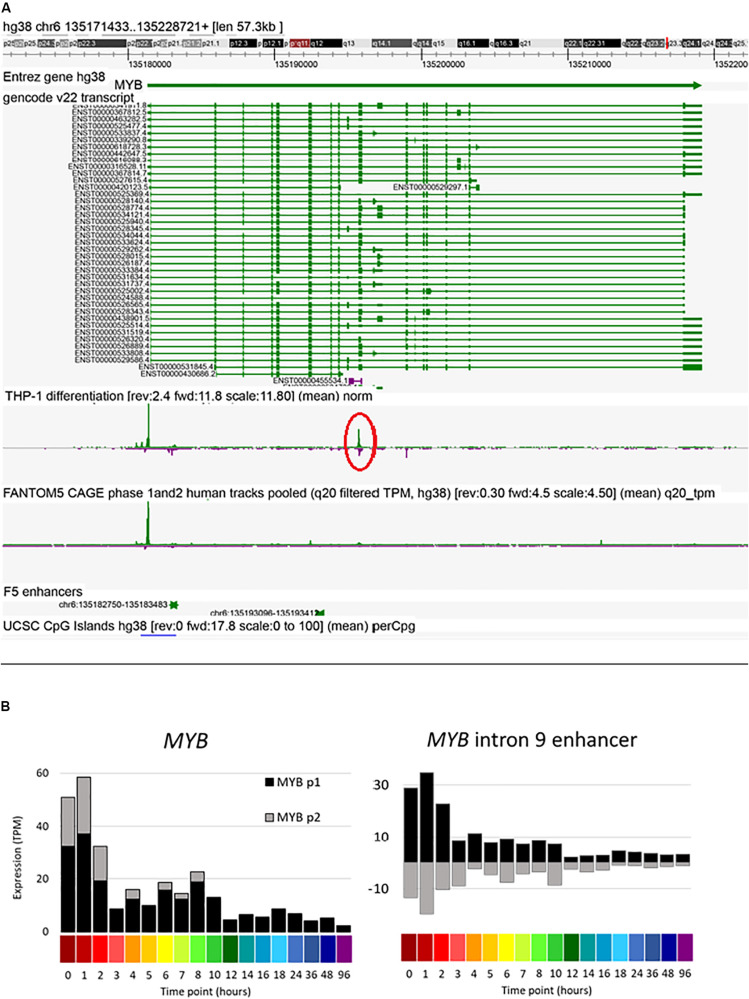
Expression of *MYB* intron 9 putative enhancer during THP-1 differentiation. **(A)** Screenshot of Zenbu browser showing *MYB* intron 9 putative enhancer (circled in red). **(B)** Expression of two *MYB* promoters and the putative intron 9 enhancer. *Y* axis shows expression levels in TPM; negative value (gray bars in panel on right) indicates expression from the reverse strand. *X* axis shows the time points (hours after administration of PMA; color code as shown in Figure 2).

Amongst other genes of interest, the *MYB* gene contains two upstream and two intronic enhancers. A candidate enhancer in the first intron was progressively down-regulated in response to PMA. This same region, conserved across species, has previously been implicated in regulated expression of *Myb* in mouse erythroleukemia cells (Suhasini et al., 1997). The data reveal a second major locus of regulated bidirectional transcription in intron 9 (Figure 7). Previous studies of regulated expression of *CDKN1A* have emphasized the impact of PMA on promoter activity and the binding of transcription factor SP1. The FANTOM5 study identified four enhancers in the region of *CDKN1A*. One of these, at Chr6:36667005-36667447 (around −12 kb relative to the TSS and conserved in mouse) was strongly and rapidly induced by PMA (Supplementary Figure S3). The progressive induction of the *CSF1R* gene in response to PMA was preceded by increased antisense transcription initiation in the first intron (Supplementary Figure S3), which contains two enhancers including the highly conserved FMS intronic regulatory element (FIRE) (Rojo et al., 2017). Finally, the FANTOM5 data identified candidate enhancers between the *ITGAM* (encoding CD11B) and *ITGAX* (CD11C) loci, both genes induced late in differentiation by PMA, and the CAGE data detect bidirectional transcription in the intergenic region (Supplementary Figure S3).

## Discussion

This study used CAGE to analyze the transcriptional regulatory networks that are initiated in response to phorbol esters in a high differentiation clone of the THP-1 myeloid leukemia line. The analysis of the cell cycle arrest is consistent with earlier reports. Using the parent line, Traore et al. (2005) found a similar proportion of cells in S phase in undifferentiated cells and reported a complete inhibition of DNA synthesis (assessed by BrdU uptake) in response to PMA within 24 h. They therefore implicated a block to G1/S entry which is also evident from the rapid loss of intermediate (S phase) DNA content in our study (Figure 1). The persistence of cells with 4N DNA content in both their study and ours indicates at least some of the cells are then blocked at the G2/M checkpoint, as also seen in AML cells treated with genotoxic agents (Didier et al., 2008).

Compared to the earlier analysis by the FANTOM4 consortium (Suzuki et al., 2009) we have analyzed a much denser time course, sequenced at greater depth using next generation sequencing platforms, and taken advantage of the depth of data to perform a network-based analysis of the temporal cascade. Minor differences between our study and FANTOM4 in detection of specific genes (e.g., *TP53, CSF1R*) are likely due to differences in sequencing technology/depth/error rates/mapping and refinement of the human genome assembly in two updates since the previous study (which was mapped to hg18, NCBI Build 36.1, released in 2006). Where there are differences, our data are validated by multiple replicates at multiple time points and the precise location of the TSS. The FANTOM5 consortium generated similar data for a number of differentiation/activation cascades in other cell types (Arner et al., 2015). Those data enabled the identification of eRNA derived from active enhancers and provided evidence that transient activation of eRNA transcription preceded the activation of target genes. The data generated for THP-1 cells complement and extend the detailed chromatin analysis published by others (Phanstiel et al., 2017), which compared only the unstimulated and fully differentiated states (after 72 h) and used the parent THP-1 line in which only a subset of cells responds to PMA. They emphasized the role of AP1 transcription factors in the formation of chromatin loops between distal enhancers and macrophage-specific promoters, a conclusion that is supported by the MARA analysis herein.

One of the cellular systems analyzed in FANTOM5, in which there was a similar extensive change in gene expression, was the activation of human monocyte-derived macrophages by bacterial lipopolysaccharide (LPS) (Baillie et al., 2017). Network-based analysis of that response identified waves of transiently induced promoters/transcripts extending across the entire time course up to 72 h. Each cluster of LPS-induced or repressed transcripts contained both candidate feedback repressors of the previous transcriptional cluster and transcription factors and autocrine growth factors that contributed to the regulation of subsequent clusters. The same pattern of transient waves of inducible transcripts is evident in this PMA time course in the high differentiation THP-1 cell line. For example, in the network analysis (Supplementary Table S1), Cluster020 contains immediate early transcription factors as well as feedback regulators such as *ZFP36*, which encodes an enzyme that degrades unstable AU-rich mRNAs. The related gene, *ZFP36L1*, was even more highly induced by PMA, peaking after 2–4 h and remaining elevated. Over-expression of ZFP36L1 was shown to positively-regulate differentiation in THP-1 cells. This was attributed to degradation of *CDK6* mRNA (Chen et al., 2015), but this transcript was actually induced by PMA.

Cell cycle arrest in THP-1 cells probably involves multiple mechanisms. THP-1 cells were reported to lack functional TP53 and indeed *TP53* mRNA was low in THP-1 cells in the FANTOM 4 and FANTOM5 data (Suzuki et al., 2009; Forrest et al., 2014). In the current study, *TP53* mRNA was readily detectable in multiple replicates and formed part of Cluster001. Consistent with functional expression of *TP53*, PMA was previously shown to induce the TP53-responsive cell cycle regulator p21WAF1/CIP1 (encoded by *CDKN1A*). Induction of both *CDKN1A* mRNA and protein in published studies was detected after 24 hrs of PMA treatment (Traore et al., 2005). Our study shows that induction of *CDKN1A* by PMA is a much earlier event and provides insights into the mechanism. Transcriptional activation of *CDKN1A* in these cells has been linked to GC-rich binding sites for SP1 in the proximal promoter (Traore et al., 2005; Pivoriunas et al., 2007). CDKN1A has also been implicated in cell cycle arrest in response to PMA in U937 myeloid leukemia cells (Vrana et al., 1998). *CDKN1A* formed part of Cluster012, and was already strongly induced 1 h after PMA addition, well in advance of cell cycle blockade and consistent with the existence of a poised initiation complex at the *CDKN1A* promoter (Espinosa et al., 2003). The FANTOM5 data reveal the presence of regulated enhancers both upstream and downstream of the *CDKN1A* promoter that were also implicated in other human cell types (Espinosa et al., 2003). *SGK1* is another TP53 target gene (You et al., 2004) that was also immediately and massively induced by PMA. Meta-analysis of many large datasets identified only a limited set of consensus TP53-dependent genes (Fischer, 2017). Amongst the most highly validated targets, *GDF15, PLK2, PLK3, BTG2, SESN1*, and *SESN2* (each of which can contribute to cell cycle arrest; Fischer, 2017; Yuniati et al., 2019) were also induced rapidly during the THP-1 time course of differentiation presented here and most likely also controlled by poised promoters, whereas *BAX, GADD45A, MDM2, RRM2B*, and *SUSD6* were constitutively expressed.

Accordingly, it is unlikely that *CDKN1A* induction provides the sole mechanism of cell cycle inhibition in PMA-stimulated THP-1 cells. The expression of retinoblastoma (*RB1*), itself a tumor suppressor gene (Dyson, 2016), and associated with G2/M cell cycle arrest (Stark and Taylor, 2004) was rapidly induced from two separate promoters by PMA. The set of inducible genes in Cluster012 also contains *ABL2* which suppresses FLT3-induced proliferation (Kazi et al., 2017) and the tumor suppressor gene *DOCK4* (Sundaravel et al., 2015). Cluster003, which peaks transiently around 6 h, contains transcripts encoding cycle-dependent kinases encoded by *CDK19* and *CDK20* which have complex functions in cell cycle regulation (reviewed in Malumbres, 2014). By association, proteins encoded by other transcripts within these clusters could also contribute to cell cycle arrest. Amongst the most highly induced transcripts in Cluster001 are candidate regulators of proliferation including the MAP kinase inhibitor *DUSP1* and *GADD45G*, which can drive growth arrest and differentiation in hematopoietic stem cells (Thalheimer et al., 2014).

The first detected response to PMA, as in other cellular state transitions, was the induction of immediate early genes. In each case, the mean gene expression increased to a peak within 1–2 h; the patterns differed depending upon how rapidly the expression decayed. As noted in the results, many IEG encode transcription factors. One key immediate early gene that is specific to myeloid lineages is *MAFB*. *MAFB* can drive monocyte commitment when transduced into progenitor cells (Gemelli et al., 2006). In the FANTOM 5 dataset, *MAFB* mRNA was most highly expressed in monocytes and macrophages and induced in the transition from committed granulocyte-macrophage progenitors (Joshi et al., 2015). In the current study, *MAFB* was an IEG, maximally up-regulated after 2 h and sustained thereafter. *MAFB* mRNA and protein were reportedly also up-regulated in THP-1 cells in response to a different differentiation signal, ATRA, in combination with tumor necrosis factor (Zhang et al., 2012). Given the rapidity of induction in our study, and the fact that the *MAFB* locus is in open chromatin in THP-1 cells (Suzuki et al., 2009; Phanstiel et al., 2017), transcriptional activation of *MAFB* most likely involves a poised initiation complex. Zhang et al. (2012) predicted target genes for *MAFB* based upon the presence of MAF-response elements (MARE) in their promoters. The MARE contains a consensus AP1 element and is partly captured in our MARA analysis. The majority of the 64 putative MAFB target genes (including *AIM2, CCL2, LPXN, PRDM1, SLC15A3, SPOCK1*) were also progressively induced in our study. However, although knockdown of MAFB compromised expression of target genes such as *PRDM1* and *SPOCK1*, it did not prevent differentiation (Zhang et al., 2012).

Several other proteins encoded by IEG, notably JUN and EGR2, also have specific functions in macrophage differentiation (Rojo et al., 2017). NR4A1 is required specifically for the maturation of blood monocytes to form the non-classical (in mice, Ly6C^–^, in humans CD16^++^) subset (Thomas et al., 2016). Deletion of part of an enhancer in the mouse *Nr4a1* locus selectively ablated expression in monocytes (Thomas et al., 2016). The FANTOM5 data reveal multiple upstream enhancers within *NR4A1* and the present THP-1 time course reveals at least two regions of inducible bidirectional promoter activity.

Whereas there are numerous DNA-modifying transcription factors up- and down-regulated across the time course of differentiation, few regulated transcripts encoding chromatin modifying enzymes were identified that could contribute to the large-scale chromatin looping reported by Phanstiel et al. (2017). The exception is *KDM6B* (also known as *JMJD3*), which was almost undetectable in unstimulated cells and rapidly induced alongside IEG transcription factors. In mice, KDM6B is required for normal macrophage differentiation (Satoh et al., 2010). Consistent with the induction during monocytic differentiation in THP-1 cells, *KDM6B* was highly expressed in human monocytes and induced further with adhesion (Forrest et al., 2014). KDM6B is also recruited to TP53-dependent promoters in many other cell types, so likely contributes also to growth arrest (Williams et al., 2014).

The large set of transcripts within Cluster001 that was progressively up-regulated across the time course of THP-1 differentiation provides a rich resource for the identification of novel genes that are involved in cell cycle arrest on the one hand and macrophage differentiation on the other. Their inferred function is implied by stringent coregulation with many genes of known function including macrophage differentiation markers such as *CD14, CD163, CSF1R*, *ITGAM*, and *MERTK* and by cross-comparison with primary cells [stem cells, committed progenitors, monocytes and monocyte-derived macrophages in the FANTOM5 data (Joshi et al., 2015)]. Cluster001 largely overlaps with TF-Cluster04 (Supplementary Table S5) which contains 47 transcription factors that likely contribute to the terminal differentiation process. Of these candidate regulators, the FANTOM5 data indicate that all but 3 (*SHOX2, JRKL, SOX4*) are expressed in human monocytes or monocyte-derived macrophages from the same transcription start sites. Amongst the potential novel regulators, *KLF6* has been implicated in mouse macrophage polarization (Kim et al., 2020) but has not previously been studied in human monocytes. Consistent with late up-regulation in THP-1 cells, in the FANTOM5 data, *KLF6* was highly expressed by monocytes and further up-regulated in response to adhesion. Induction of *KLF6* in THP-1 cells was associated with activation of a cluster of enhancers detected by bidirectional transcription initiation 20–50 kb upstream that overlap monocyte-enriched enhancers detected in the FANTOM5 data.

CAGE data also enables analysis of the precise transcription start site used in the cell line compared to primary cells. For example, as shown in Figure 6, *TPRG1* (TP63-regulated gene 1) was highly induced in parallel with *BCL6*. *BCL6* is highly expressed by human monocytes (Forrest et al., 2014). The original description of the mouse gene *Tprg1* noted the location immediately upstream of *Trp63* and described expression specifically in the skin and down-regulation following a *Trp63* knockdown (Antonini et al., 2008). This region on mouse chromosome 16 is syntenic with the region of the human *TPRG1* gene on chromosome 3. The TPRG1 protein function is unknown. In the FANTOM5 data, *TP63* is expressed specifically in epithelial cells from multiple locations, but it was barely detectable in baseline THP-1 cells. By contrast its role in developmental regulation in the mouse (Antonini et al., 2008), *TPRG1* expression was not correlated with *TP63* in the human transcriptome (Forrest et al., 2014). It was most highly expressed in adherent monocytes and monocyte-derived macrophages. Like many human genes (Forrest et al., 2014), *TPRG1* has multiple tissue-specific promoters and THP-1 and monocytes share the use of the most distal transcription start site generating a unique non-coding 5′ exon. Similarly, *FRMD4B* was highlighted as a transcript induced in parallel with *MITF* and likely sharing enhancers. The parallel induction of these neighboring genes was also observed in the generation of monocyte-derived macrophages in response to CSF1 in the FANTOM5 data. Like primary human monocytes and macrophages, THP-1 cells initiated transcription of *MITF* primarily from the most 5′ of at least 6 tissue-specific promoters. *FRMD4B* also has multiple transcription starts sites and THP-1 and monocyte-derived macrophages each initiate transcription from the most 5′ location. The only previous functional analysis of FRMD4B protein (also known as GRSP1) described an interaction with GRP1 (the product of *CYTH3*), a protein involved in regulation of cell adhesion (Klarlund et al., 2001). One other example of novel co-regulated genes is the *ADCY8/ASAP1* locus on chromosome 8. Both transcripts were rapidly induced by PMA and there was extensive, inducible bidirectional transcription initiation between the two genes, as well as within and upstream of *ADCY8* to the *EFR3A* gene, corresponding also to FANTOM5 enhancers. *ADCY8* encodes a calcium-sensitive adenylate cyclase that has previously been shown to have brain-specific neuronal functions (Masada et al., 2009) and the brain-specific expression is confirmed in the FANTOM5 data. *ASAP1* encodes ARFGAP with SH3 domain, ankyrin repeat and PH domain 1. This transcript is also a differentiation marker in primary human cells; up-regulated in monocyte-derived macrophages compared to blood monocytes in the FANTOM5 data. Like FRMD4B the ASAP1 protein product is likely involved in cytoskeletal function in the development of adhesion (Liu et al., 2002).

Conversely, in parallel with the loss of cell cycle-associated transcription, the set of transcripts that is down-regulated progressively in response to PMA includes genes that are specific to monocytes and repressed upon macrophage differentiation. One such gene is *IRF8. IRF8* mutations in humans are associated with monocyte deficiency (Crosslin et al., 2013). Other monocyte-specific transcripts down-regulated by PMA include the chemokine receptor, *CCR2*, the receptor for granulocyte colony-stimulating factor (*CSF3R*), the tetraspanin *MS4A3* (recently identified as a marker of monocytic progenitors; Liu et al., 2019), P selectin ligand (*SELPLG*), surface markers (e.g., *FCGR2A, CD38, CD302, CD320*, *GPR160*) and *S100A8/S100A9*, encoding the cytoplasmic calgranulin complex. *MS4A3* is expressed by committed human myeloid progenitors and down-regulated in monocytes (Forrest et al., 2014). *CSF3R, CCR2, CD302*, and *S100A8/9* are amongst the large set of genes down-regulated during differentiation of macrophages from blood monocytes (Baillie et al., 2017).

## Conclusion

This study describes a detailed time course of growth arrest and differentiation of the AML line, THP-1. Whereas these processes are commonly portrayed as linear sequences with a beginning and an end point, the time course reveals a sequential cascade of transient changes in gene expression. Even at 96 h post-stimulation, there are macrophage-associated genes that appear to be increasing their expression (such as CD14), most likely still responding to signals from CSF1R and adhesion to the substratum. In mixed population of monocyte-macrophages in tissues, differentiating asynchronously, this sequential cascade might be (mis)interpreted as evidence of the existence of distinct monocyte subpopulations. Hundreds of signaling molecules, transcription factors and feedback regulators are involved. The analysis reveals many novel inducible genes (e.g., *TPRG1, FRMD4B*) for which there is currently no functional annotation. THP-1 has been studied as an archetype of the subset of AML that can respond to differentiation therapy (Tagliafico et al., 2006). A detailed understanding of the process of differentiation and growth arrest may highlight new therapeutic targets and opportunities.

## Data Availability Statement

The datasets generated for this study can be found at http://fantom.gsc.riken.jp/zenbu/gLyphs/#config=Gazova_THP1_differentiation. The raw data files have been uploaded to the European Nucleotide Archive, BioProject ID PRJEB38762.

## Author Contributions

IG performed the cell culture, CAGE analysis and validation, and wrote the first draft of the manuscript. LL was involved in CAGE library preparation. SB and SC helped with the bioinformatic analysis of CAGE sequencing results. EA performed the MARA analysis. MH and JS created the visualization platform and helped prepare the data for visualization. LD and RA performed the enhancer analysis. AL was involved in interpretation of the results and supervision. DH and KS were responsible for conception and design of the project, analyzed the data, wrote the manuscript, and supervised the project. All authors approved the submitted version.

## Conflict of Interest

The authors declare that the research was conducted in the absence of any commercial or financial relationships that could be construed as a potential conflict of interest.

## Footnotes

1 Now BioLayout, available at http://biolayout.org

2 http://david.ncifcrf.gov

3 https://github.com/anderssonlab/CAGEfightR_extensions

4 http://fantom.gsc.riken.jp/zenbu/gLyphs/#config=Gazova_THP1_differentiation

## Supplementary Material

FIGURE S1 qPCR results during differentiation for MYB and CD14 (includes full time course with intermediate points not sampled for CAGE).

FIGURE S2 Histograms of transcription factor motif activity during THP-1 differentiation.

FIGURE S3 Enhancers for *CDKN1A, CSF1R, ITGAM* and *ITGAX*.

TABLE S1 List of clusters and average expression patterns of genes during THP-1 differentiation.

TABLE S2 List of clusters and average expression patterns of cell cycle subclusters.

TABLE S3 List of clusters and average expression patterns of curated cell cycle genes.

TABLE S4 List of curated cell cycle genes not detected in THP-1 cells undergoing differentiation.

TABLE S5 List of clusters and average expression patterns of curated transcription factors.

TABLE S6 Transcription factor motif activity (MARA).

TABLE S7 Comparison between enhancers in the THP-1 time course and enhancers in the main FANTOM5 set.

FILE S1 Full protocol for creation and quality control of CAGE libraries.
