# Supplementary material for: The Transcriptional Network That Controls Growth Arrest and Macrophage Differentiation in the Human Myeloid Leukemia Cell Line THP-1
Source: Front Cell Dev Biol. 2020 Jul 3;8:498. doi: 10.3389/fcell.2020.00498 (PMC7347797; doi:10.3389/fcell.2020.00498)

Figure S1. Quantitative reverse transcriptase PCR results for proliferation marker *MYB* and macrophage marker *CD14.* Y axis shows expression normalised to *ACTN.* X axis shows time points in the THP-1 differentiation series. There were two technical replicates per sample and 3 to 6 samples per time point. Note that this analysis includes all time points in the analysis, with additional time points between 0 and 4 hours, consistent with the time courses discussed presented in the FANTOM5 data at http://fantom.gsc.riken.jp/zenbu/.

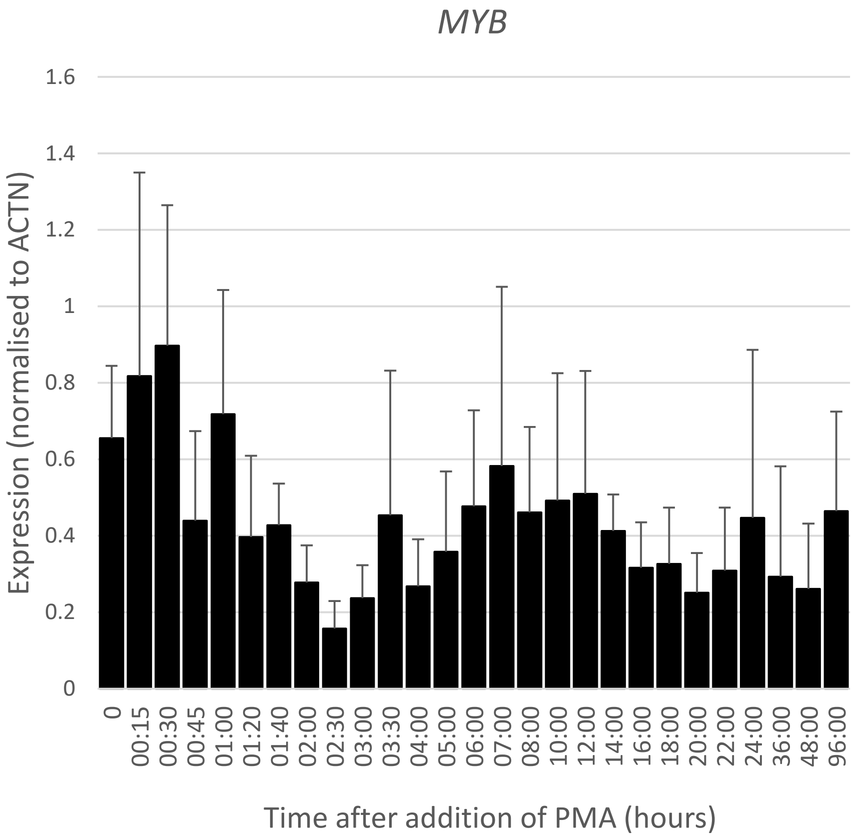

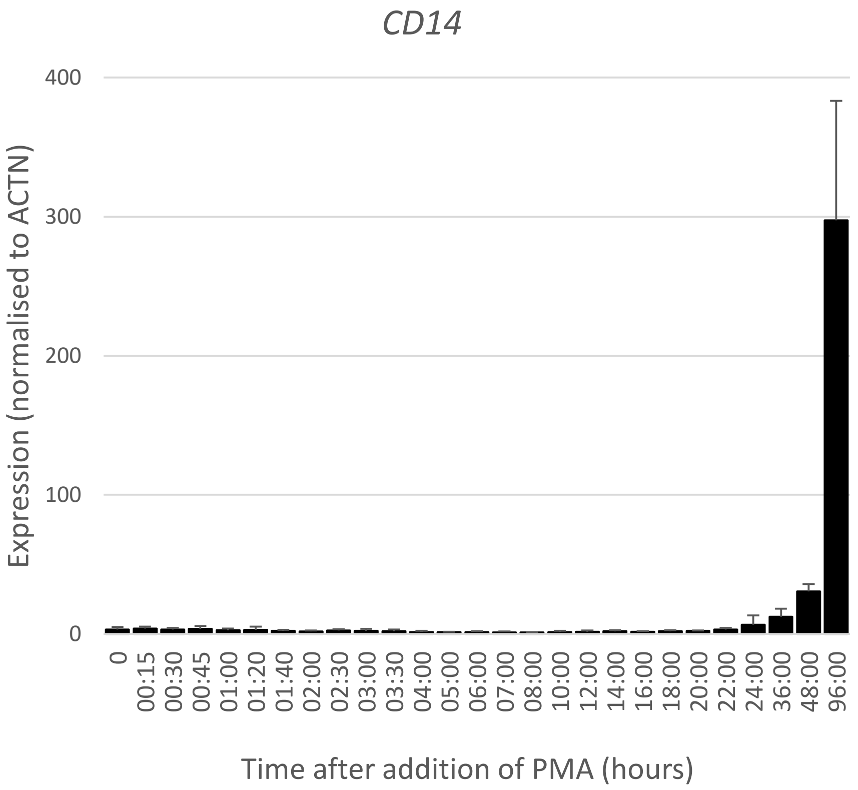

Figure S2. Transcription factor motif activity during THP-1 differentiation. Activity is averaged for each time points (2 to 6 replicates). Time points are as described in the text with the same colour coding.

A. Motifs with increasing activity.

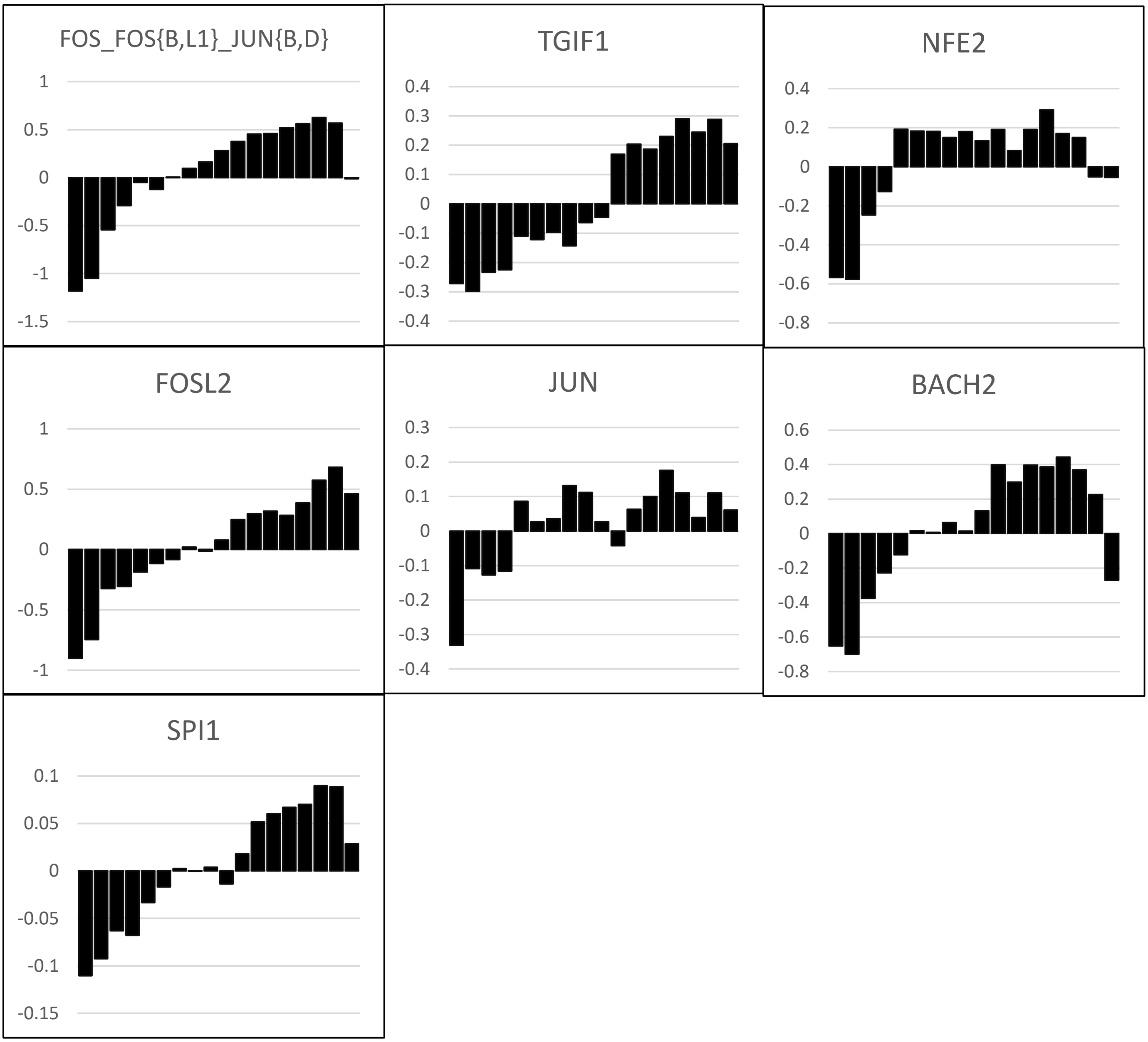

B. Motifs with transient activity.

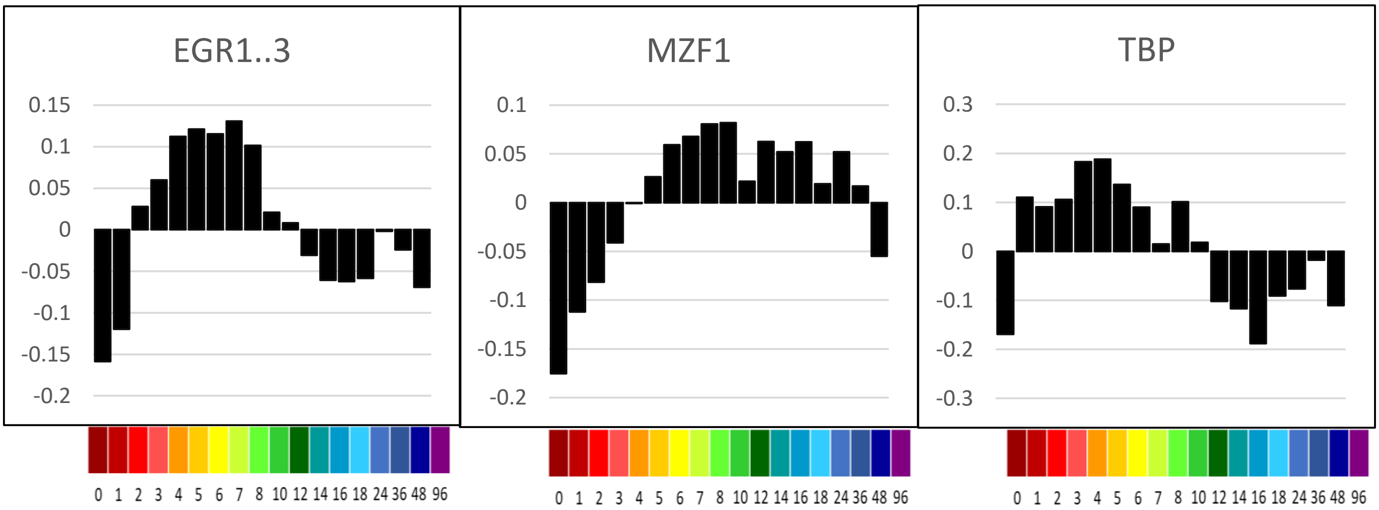

Figure S2 (continued). Transcription factor motif activity during THP-1 differentiation (continued). Activity is averaged for each time points (2 to 6 replicates).Time points are as described in the text with the same colour coding.

C. Motifs with decreasing activity.

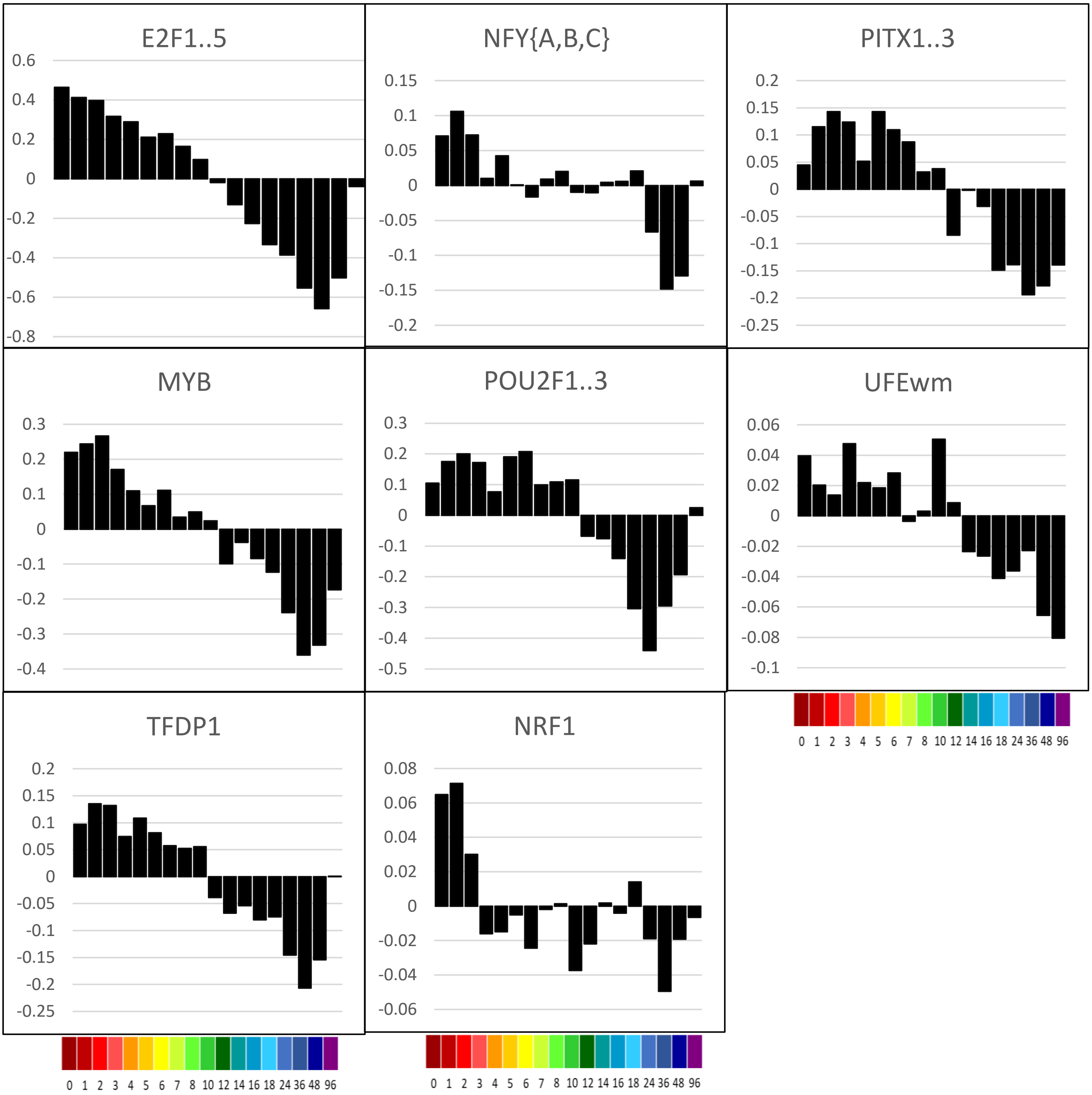

Figure S3. Enhancers associated with key genes during THP-1 differentiation

A. *CDKN1A*

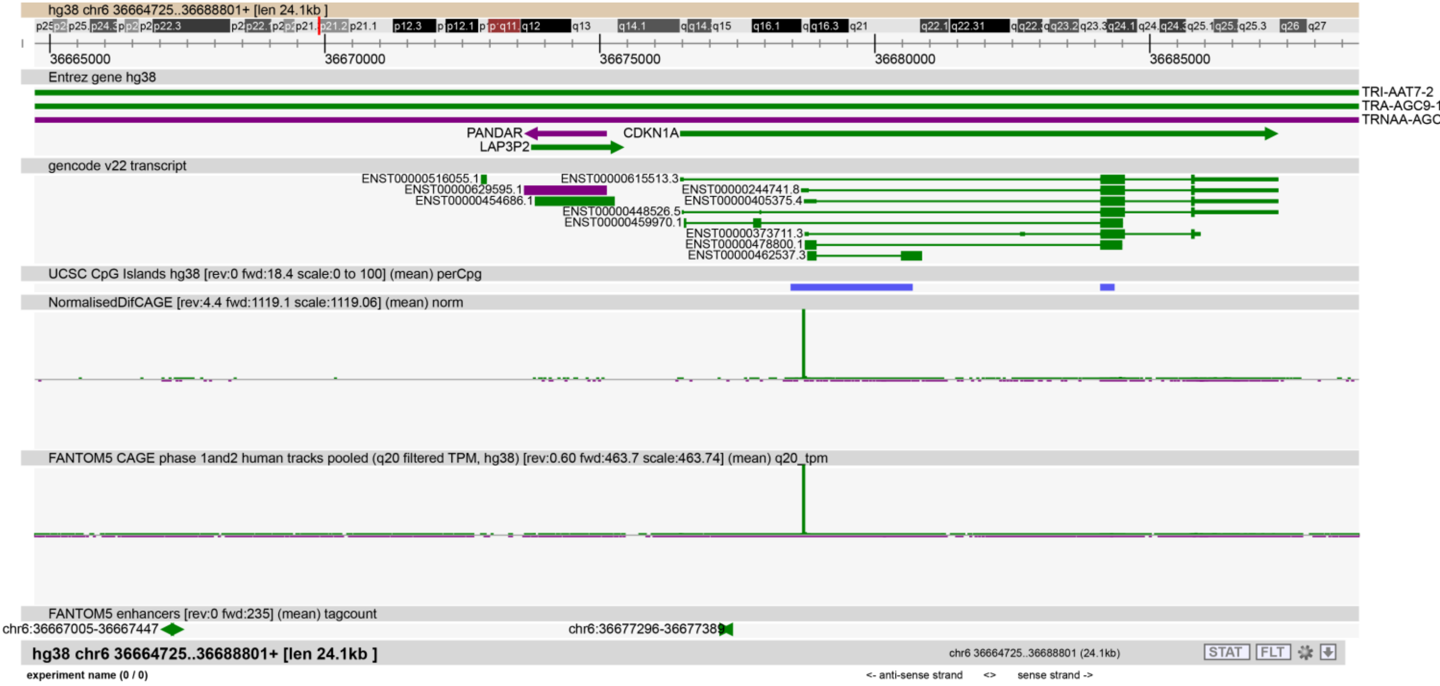

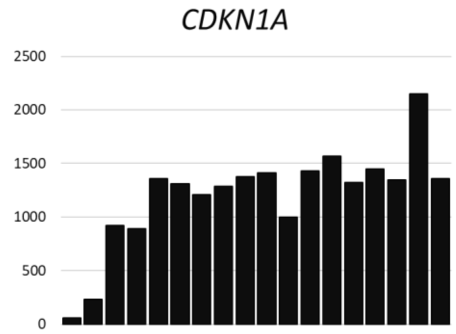

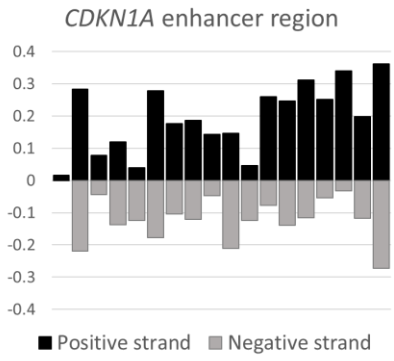

Figure S3. **E**nhancers associated with key genes during THP-1 differentiation (continued)

B. *CSF1R*

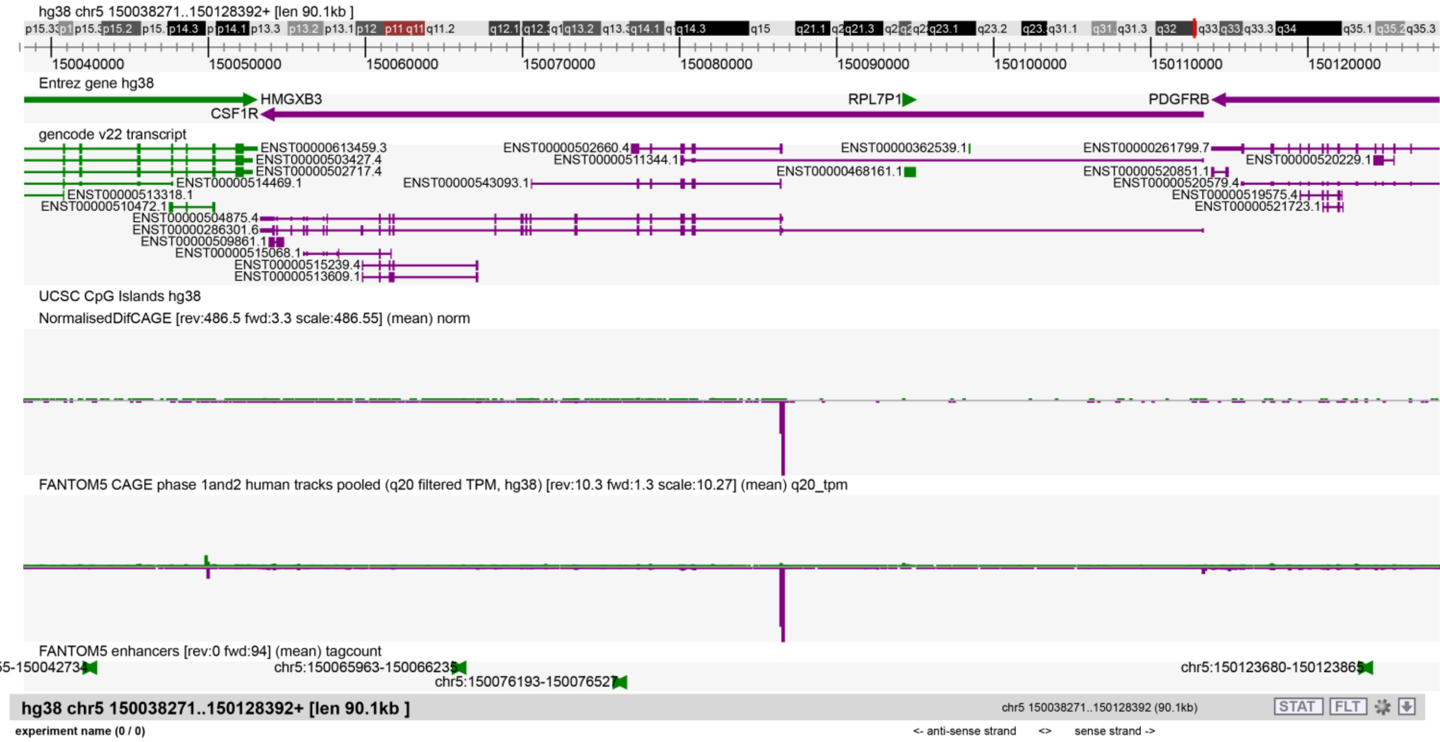

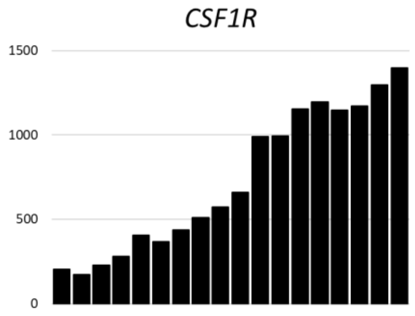

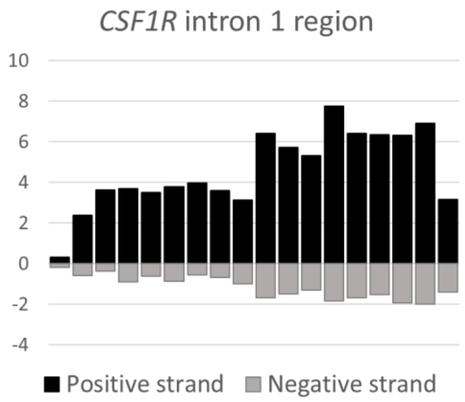

Figure S3. Enhancers associated with key genes during THP-1 differentiation (continued)

C. *ITGAM and ITGAX*

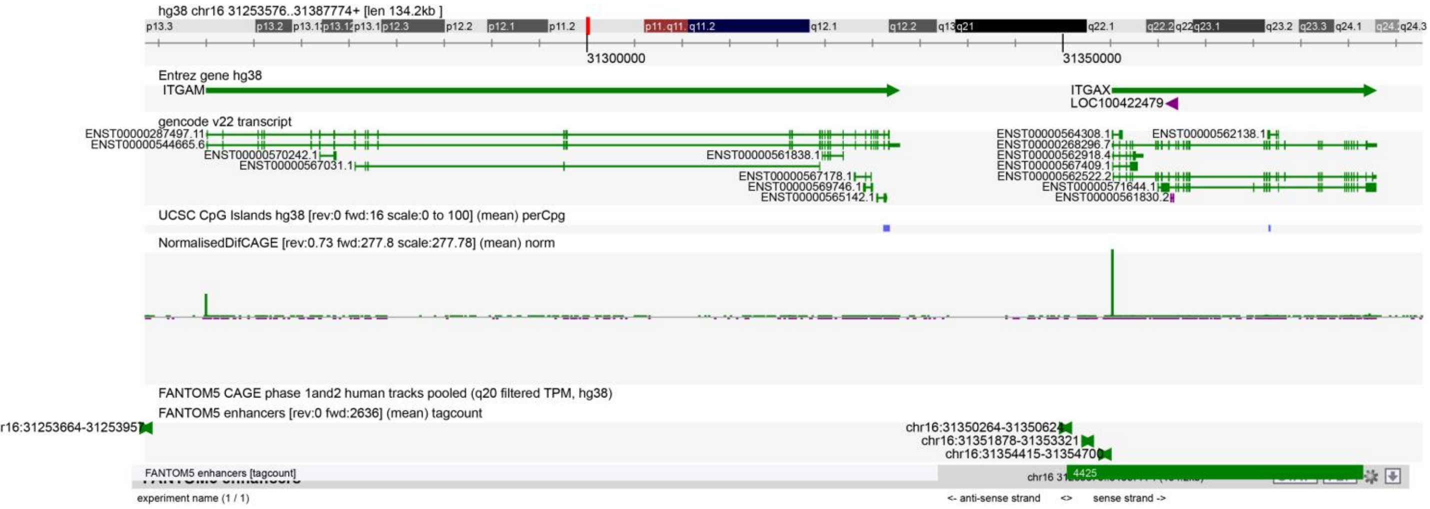

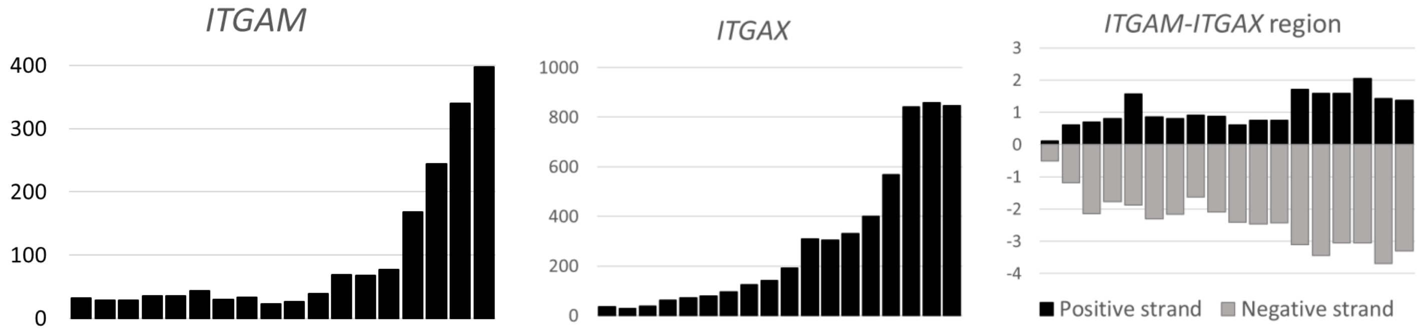

Supplement: FIGURE S1 — qPCR results during differentiation for MYB and CD14 (includes full time course with intermediate points not sampled for CAGE). [file Data_Sheet_2.PDF]
