# Supplementary material for: The Transcriptional Network That Controls Growth Arrest and Macrophage Differentiation in the Human Myeloid Leukemia Cell Line THP-1
Source: Front Cell Dev Biol. 2020 Jul 3;8:498. doi: 10.3389/fcell.2020.00498 (PMC7347797; doi:10.3389/fcell.2020.00498)
Supplement: FILE S1 — Full protocol for creation and quality control of CAGE libraries. [file Data_Sheet_1.PDF]

Supplementary Materials

1. SELECTION OF RNA SAMPLES FOR CAGE LIBRARIES

RNA samples from the time courses were assessed for quality (RIN^e score) and quantity (concentration). Taking together all the information, 66 samples were selected for CAGE library preparation. The lowest RIN^e score for samples included in the analysis was 7.7; the majority of samples had RIN^e score of > 9.5.

2. CAP ANALYSIS OF GENE EXPRESSION (CAGE) METHODOLOGY

The procedure is summarised in the diagram below**.**

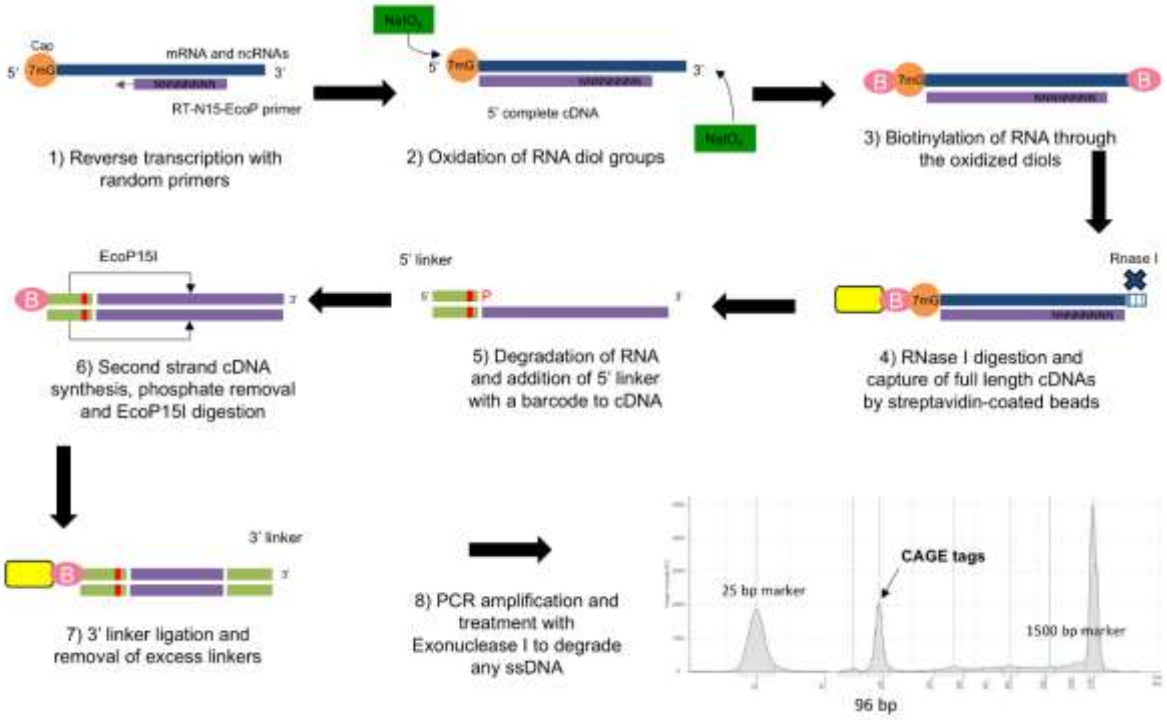

2.1 Overview of CAGE library preparation.

Total RNA from cells is reverse transcribed with random primers with an *Eco*PI site (1). Next, $NaIO_4$ oxidises diol groups (2) to open them so the biotin can attach to the cap (3). The ssRNA on the ends is digested and the biotin-labelled cap with cDNA/RNA hybrid is captured by streptavidin beads (4). The cap is deattached, the RNA is digested, and 5' linker with a barcode and *Eco*P15I site is attached (5). Second strand of cDNA synthesized and prior to endonuclease digestion, phosphates are removed by Antarctic phosphatase to prevent nonspecific binding of 3'linker in the further step (6). *Eco*P15I cleaves 27 nt downstream, leaving only the 5' linker with 27 nt of cDNA, to which the 3' end of another linker is attached. Excess linkers need to be removed by vigorous washing steps (7), otherwise PCR amplification picks up high levels of linker DNA (8). The lowest possible number of PCR cycles is chosen (from 9 to 11, usually 10) to reduce PCR bias (8).

2.2 Details of CAGE library preparation.

Eight samples of 5 µg RNA each were made into one CAGE library following the protocol adapted from (Takahashi et al., 2012). All the primer sequences were taken from (Takahashi et al., 2012). A list of oligonucleotide sequences is given in the table below. They were synthesized by IDT (Coralville, IA, USA) and prepared according to (Takahashi et al., 2012).

5 µg of RNA per sample, concentrated to 5.3 µl using a vacuum concentrator (Speed Vac, RC 10.22, Jouan SA, Saint-Herblain, France), was mixed with 2.2 µl of 210 µM (2 µg/µl) RT-N15-*Eco*PI primer (7.5 µl total) and incubated at 65 °C for 5 min and then cooled on ice immediately. The RNA with primer was reverse transcribed to a cDNA/RNA duplex by mixing the components detailed in Table 2 2, and incubated at 25 °C for 30 s, 42 °C for 30 min, 50 °C for 10 min, 56 °C for 10 min, and 60 °C for 10 min.

2.3 Reverse transcription of RNA (Step 1)

Component	Volume (µl)	Final concentration
PrimeScript buffer (Takara Clontech, Otsu, Japan)	7.5	1X
dNTPs (10 mM each, Invitrogen)	1.87	0.5 mM each
Sorbitol (3.3 M)/(0.66 M) trehalose mix solution	7.5	0.66 M/0.132 M
PrimeScript™ reverse transcriptase (200 U/µl; Takara Clontech)	3.75	750 U
Water	9.38	-
Total Volume	30	-

Then, cDNA/RNA hybrids were purified with Agencourt® RNAClean® XP beads (ratio 1.8 to 1; Beckman Coulter, Indianapolis, IN, USA) according to manufacturer's instructions. The beads with the samples were incubated at room temperature for 30 minutes, mixing 10 times every 10 minutes. The beads were separated from the supernatant using a 96-well magnetic stand (DynaMag® 96-slide, Life Technologies, USA), and washed twice with 150 µl of 70% ethanol. Then, 40 µl of water heated to 37 °C for elution was added and mixed thoroughly. Incubation at 37 °C lasted 5 minutes minimum. The eluate with the cDNA/RNA hybrids was separated from the beads using the magnetic stand and transferred to a new tube.

Next, the cDNA/RNA hybrid was diol oxidated with 250 mM $NaIO_4$ (0.053 g of $NaIO_4$ in 1 ml of water; Sigma-Aldrich). This step oxidates the 7-methyl-guanylate (cap) on the 5' end of the RNA as well as the 3' end of the RNA. The reagents were mixed and incubated on ice for 45 minutes in darkness.

2.4 Oxidation of RNA diol groups (Step 2)

Component	Volume (µl)	Final concentration
RNA-cDNA hybrid	40	-
Sodium acetate (1 M, pH 4.5, sterile-filtered)	2	45.7 mM
$NaIO_4$ (250 mM)	2	11 mM
Total volume	44	-

After incubation, 2 µl of 40% (wt/vol) glycerol (Fischer Scientific, Waltham, MA, USA) was added to stop the oxidation reaction. 14 µl of 1 M Tris-HCl (pH 8.5, sterile-filtered) was mixed in to bring the pH above 5.6 (total volume 60 µl). Then, cDNA/RNA hybrids were purified again with Agencourt® RNAClean® XP beads as above.

The RNA diols were then biotinylated with 15mM of biotin hydrazide (long arm; 0.0038g in 675µl of water; Vector Laboratories, Burlingame, CA, USA) by mixing the reagents (below) and incubating at 23°C overnight. This step adds a biotin to the diol oxidised cap.

2.5 Biotinylation of RNA (Step 3)

Component	Volume (µl)	Final concentration
Purified oxidated cDNA/RNA hybrids	40	-
Sodium citrate (1M, pH 6.0, sterile-filtered)	4	70mM
Biotin hydrazide (long arm, 15mM)	13.5	3.5mM
Total volume	57.5	-

The next day, the biotinylated diols were subjected to RNase ONE ribonuclease treatment by mixing the reagents (below) and incubated at 37 °C for 30 minutes and 65 °C for 5 minutes. This degrades all single stranded RNA segments. Only cDNA/RNA heteroduplexes will remain, and only those containing the 5' cap (that is the 5' end of the RNA) will be biotinylated.

2.6 RNase digestion (Step 4 and 5)

Component	Volume (µl)	Final concentration
Biotinylation reaction	57.5	-
Tris-HCl (1 M, pH 8.5, sterile-filtered)	6	86 mM
EDTA (0.5 M, pH 8.0, Ambion)	1	7.2 mM
RNase ONE ribonuclease (10 U/µl, Promega)	5	50 U
Total volume	69.5	-

Then, cDNA/RNA hybrids were purified with Agencourt® RNAClean® XP beads as above.

MPG® Streptavidin beads (Takara Clontech) were prepared by mixing 100µl of beads per sample and 1.5 µl of tRNA (20 µg/µl; transfer ribonucleic acid from *Escherichia coli*, Sigma-Aldrich) per sample at 4 °C for at least an hour by continuous mixing. Beads were then separated from the supernatant using a 96 well magnetic stand (SureBeads® BioRad, USA) and washed twice with 50 µl per sample of wash buffer 1 (45 ml of 5 M NaCl and 5 ml of 0.5 M EDTA pH 8). Finally, they were resuspended in 80 µl of wash buffer 1 per sample.

The streptavidin-tRNA-coated beads were used to trap the biotinylated RNA/cDNA heteroduplexes. The purified sample was mixed with 80 µl of the MPG® Streptavidin beads coated with tRNA (120 µl total) and incubated at room temperature for 30 minutes, mixing 10 times every 5 minutes. The beads were separated from the supernatant using the 96-well magnetic stand and washed 6 times total with various buffers: once with 150 µl of wash buffer 1, once with 150 µl of wash buffer 2 (3 ml of 5 M NaCl, 100 µl of 0.5 M EDTA pH 8, and 46.9 ml water), twice with 150 µl of wash buffer 3 (1 ml of 1M Tris-HCl (pH 8.5), 100 µl of 0.5M EDTA (pH 8), 25 ml of 1 M sodium acetate (pH 6.1), 2 ml of 10% (w/v) SDS and 21.9 ml of water), and twice again with 150 µl of wash buffer 4 (500 µl of 1 M Tris-HCl (pH 8.5), 100 µl of 0.5 M EDTA (pH 8), 25 ml of 1 M sodium acetate (pH 6.1), and 24.4 ml water).

RNA was degraded and the cDNA released from the beads (**(Step 5, Supplementary Figure 1)** by adding 60 µl of 50 mM NaOH solution and incubating at room temperature for 10 minutes, pipetting to mix every 2-3 minutes. The solution was then buffered to a lower pH by adding 12 µl of ice cold 1 M Tris-HCl pH 7.0 (Trizma® hydrochloride, Sigma-Aldrich).

Then, the single stranded cDNA was purified with Agencourt® AMPure® XP beads (1.8 volume of beads to 1 volume of sample; Beckman Coulter) according to manufacturer’s instructions. The beads with the samples were incubated at room temperature for 30 minutes, mixing 10 times every 10 minutes.

The beads were separated from the supernatant using the 96-well magnetic stand, and washed twice with 150 µl of 70% ethanol. Then, 40 µl of preheated 37°C water for elution were added and mixed thoroughly. Incubation at 37 °C lasted 5 minutes minimum. The eluents with cDNA were separated from the beads by magnetic stand and transferred to a new tube.

The cDNA was then concentrated to 4 µl at room temperature using a centrifugal vacuum concentrator as described previously. The cDNA was incubated at 65 °C for 5 min and cooled on ice for 2 min. In a separate tube, 1.5 µl of the 5’ linker per sample was incubated at 37 °C for 5 minutes and cooled on ice for 2 minutes minimum. Afterwards, 1 µl of the linker was added to the cDNA and mixed with 10 µl of DNA Ligation Kit Mighty Mix (Takara Clontech) and together they were incubated at 16 °C overnight **(Step 5)**.

The next day, the samples were purified twice using Agencourt® AMPure® XP beads as above. Up to 4 samples could be pooled in 1 tube in the first purification step and 8 total in the second. In the first step, 4 samples were pooled in 1 tube (60 µl) and 10 µl of water was added (total volume 70 µl; for 8 samples there were 2 tubes of 70 µl). Then, cDNA was purified with Agencourt® AMPure® XP beads as above.

This process was repeated for the second time, but at the first was step, 150 µl of 70% ethanol was used to wash both of the wells and the contents were mixed. The samples were eluted in 30.5 µl of preheated 37°C water.

The second stand was then synthesized using La Taq Polymerase, by adding the reagents listed below and incubating at 94 °C for 3 minutes, 42 °C for 5 minutes, 68 °C for 20 minutes and 72 °C for 2 minutes. The primer for this synthesis was biotinylated.

2.7 Second strand cDNA synthesis (Step 6)

Component	Volume (µl)	Final concentration
10X La Taq buffer (TAKARA Clontech)	5	1X
$MgCl_2$ (25 mM, TAKARA Clontech)	5	2.5 mM
dNTPs (2.5 mM, TAKARA Clontech)	8	0.4 mM each
2nd SOL primer (200 ng/µl)	1	2.4 µM
La Taq Polymerase (5 U/µl, TAKARA Clontech)	0.5	2.5 U
Total volume	19.5	-

A phosphate group was then removed from the 5’ lower linker of the cDNA by adding 6 µl of 10X Antarctic Phosphatase Reaction Buffer and 4 µl of Antarctic Phosphatase (5 U/µl; New England Biolabs (NEB), Hitchin, Herts, UK). The mixture was then incubated at 37 °C for 60 minutes and 65 °C for 5 minutes. Then, cDNA was again purified with Agencourt® AMPure® XP beads (as above).

The cDNA was digested by *Eco*P15I restriction endonuclease (NEB) which binds to a recognition site in the second strand primer and cuts 27 nucleotides into the cDNA. The reagents below were mixed, and 10 µl of the master mix were added to 30 µl of the sample. The sample was incubated for 3 hours at 37 °C and then put on ice for at least 2 minutes.

2.8 *Eco*P151 digestion (Step 6)

Component	Master Mix (enough for 10 tubes, µl)	Final concentration in sample
Water 11		
10X NEB buffer 3.1 (NEB)	40	1X
100X BSA (NEB)	4	10X
10X ATP (10 mM, NEB)	40	1 mM
Sinefungin (10 mM, Sigma-Aldrich)	4	0.1 mM
*Eco*P15I (10 U/µl, NEB)	1	1 U

After 3 hours, 1 µl of $MgCl_2$ 0.4M was mixed in and incubated at 65 °C for 20 minutes, then 4 °C for 2 minutes. Next, the 3' linker was ligated by mixing 39 µl of the reagents listed below and incubating at 16 °C for 14-16 hours overnight.

2.9 Linker ligation (Step 7)

Component	Volume (µl)	Final amount
5X 3' linker ligation buffer		
16		
5X 3' linker (100 ng/µl)	1	100 ng
T4 DNA ligase (400 U/µl, NEB)	3	1200 U
Water	19	-
Total volume	39	-

2.10 5 x 3' linker ligation buffer wash (Step 7)

Component	Volume (µl)
Tris-HCl (1 M, pH 7.0, Trizma® hydrochloride, Sigma-Aldrich)	50
ATP (10 mM, NEB)	100
BSA (10 mg/ml, NEB)	0.5
Water	49.5

The next day, 15 µl of MPG® Streptavidin beads (Takara Clontech) were incubated on ice with 1.5 µl of tRNA (20 µg/µl) for 30 minutes, mixing them every 5 minutes. The supernatant was separated from the beads using the magnetic stand, and the beads washed with 75 µl of wash buffer 1 twice. After 2 washes, the beads were resuspended in 37.5 µl of wash Buffer 1.

The samples were further purified from the 3' linker by taking advantage of the biotinylated second strand primer. 25 µl of the prepared MPG® beads with tRNA were added to 80 µl of the sample from the day before and incubated at room temperature for 30 minutes, mixing every 5 minutes. The beads were separated from the supernatant using 96-well magnetic stand and washed 7 times total with various buffers: once with 150 µl of Wash buffer 1, once with 150 µl of Wash buffer 2, twice with 150 µl of Wash buffer 3, and twice again with 150 µl of Wash buffer 4. Final wash consisted of 50 µl of water. Then the beads were resuspended in 20 µl of water and they were used as the PCR template.

A PCR reaction was carried out to find out how many cycles of PCR were needed to maximise the 96 bp product and minimise the amount of linkers (around 70 bp length). One reaction mix was prepared as below, and 2 µl of the beads were added to the mix.

2.11 PCR amplification (Step 8)

Component	Volume (µl)	Final concentration
Water 32.5		
5 x HF buffer (Thermo Scientific)	10	1X
dNTPs (2.5 mM, Invitrogen)	4	0.2 mM
Forward primer (100 µM)	0.5	1 µM
Reverse primer (100 µM)	0.5	1 µM
Phusion Polymerase (2 U/µl, Thermo Scientific or NEB)	0.5	1 U
Total	48 µl	-

The reaction was incubated in the PCR machine at 98 °C for 30 s, then 20 cycles of 98 °C 10s and 60 °C 10 s. The machine was paused after 8, 10, 12, 14 and 16 cycles to take 4µl quickly out of the tube.

Subsequently, the optimum PCR cycle number was determined by D1000 ScreenTape (Agilent Technologies). The ladder was prepared mixing 3 µl D1000 Sample Buffer with 1 µl D1000 ladder, and the samples were prepared by mixing 3 µl D1000 sample buffer with 1 µl of the cDNA sample that had been removed at different cycle numbers. The samples were vortexed at 2000 rpm for 1 minute, centrifuged, and then analysed on the Agilent 2200 TapeStation system. The lowest number of samples to give an adequate amount of product was between 9 and 11, usually 10. The amount of final PCR product (at around 97 bp) was assessed using the Agilent TapeStation, as shown in a typical graph below. All libraries had a smaller peak at around 75 bp, which was deemed to be primer dimer contamination. Representative graph shows the TapeStation result for CAGE library 5, after 10 cycles of PCR. Lower and upper markers used by the system to estimate fragment lengths are shown. CAGE tags are the longest product peak, at around 97 bp. Arrow indicates a smaller peak at 75bp, probably arising from primer dimer contamination.

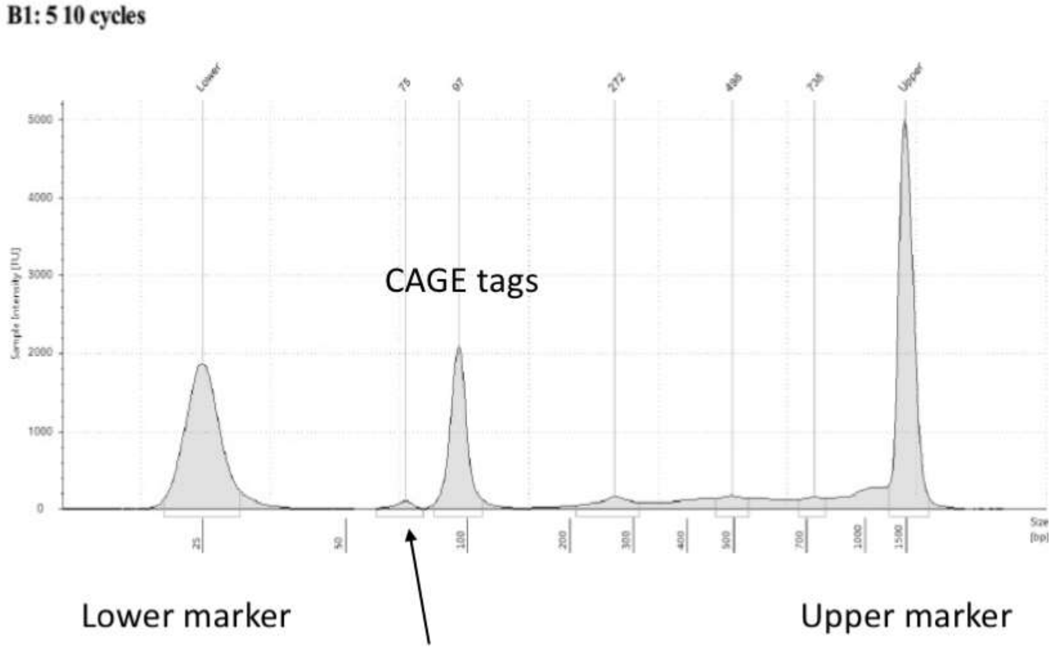

Afterwards, the PCR with the optimal number of cycles was repeated 6 times to get more product. Then, three reactions were pooled (150 µl in total) and 1 µl of Exonuclease I (20 U/µl, NEB) was added to the 150 µl PCR solution. The sample was incubated at 37°C for 30 minutes. The Exonuclease I-treated CAGE tags were purified using Minelute PCR purification kit (Qiagen), following the manufacturer's instructions. At the end, CAGE tags were eluted in 12 µl EB buffer each

and the samples pooled. Final volume was 24 µl. Quantity and quality of the tags was measured using D1000 ScreenTape as described previously.

The libraries were then sent to Edinburgh Genomics (Edinburgh, UK), where they were sequenced on an Illumina HiSeq 2500 machine (Illumina) in high throughput mode. One library (made out of eight pooled samples; seven samples in one case) was sequenced on one lane, with custom sequencing primer and inline barcodes.

2.12 Oligonucleotides used in CAGE library preparation.

Reverse transcriptase primer:

RT-N15-EcoP primer AAGGTCTATCAGCAGNNNNNNNNNNNNNNN

5’ linkers with barcodes:

5’SOL- N6-**ACG** CCACCGACAGGTTCAGAGTTCTACAGACGCAGCAGNNNNNN Phos
5’SOL- GN5-ACG CCACCGACAGGTTCAGAGTTCTACAGACGCAGCAGGNNNNN Phos
5’SOL-lower-ACG Phos CTGCTGCGTCTGTAGAACTCTGAACCTGTCGGTGG NH2

5’SOL- N6-**GAT** CCACCGACAGGTTCAGAGTTCTACAGGATCAGCAGNNNNNN Phos
5’SOL- GN5-GAT CCACCGACAGGTTCAGAGTTCTACAGGATCAGCAGGNNNNN Phos
5’SOL-lower-GAT Phos CTGCTGATCCTGTAGAACTCTGAACCTGTCGGTGG NH2

5’SOL- N6-**CTT** CCACCGACAGGTTCAGAGTTCTACAGCTTCAGCAGNNNNNN Phos
5’SOL- GN5-CTT CCACCGACAGGTTCAGAGTTCTACAGCTTCAGCAGGNNNNN Phos
5’SOL-lower-CTT Phos CTGCTGAAGCTGTAGAACTCTGAACCTGTCGGTGG NH2

5’SOL- N6-**ATG** CCACCGACAGGTTCAGAGTTCTACAGATGCAGCAGNNNNNN Phos
5'SOL- GN5-ATG CCACCGACAGGTTCAGAGTTCTACAGATGCAGCAGGNNNNN Phos
5’SOL-lower-ATG Phos CTGCTGCATCTGTAGAACTCTGAACCTGTCGGTGG NH2

5’SOL- N6-**GTA** CCACCGACAGGTTCAGAGTTCTACAGGTACAGCAGNNNNNN Phos
5’SOL- GN5-GTA CCACCGACAGGTTCAGAGTTCTACAGGTACAGCAGGNNNNN Phos
5’SOL-lower-GTA Phos CTGCTGTACCTGTAGAACTCTGAACCTGTCGGTGG NH2

5’SOL- N6-**GCC** CCACCGACAGGTTCAGAGTTCTACAGGCCCAGCAGNNNNNN Phos
5’SOL- GN5-GCC CCACCGACAGGTTCAGAGTTCTACAGGCCCAGCAGGNNNNN Phos
5’SOL-lower-GCC Phos CTGCTGGGCCTGTAGAACTCTGAACCTGTCGGTGG NH2

5’SOL- N6-**TAG** CCACCGACAGGTTCAGAGTTCTACAGTAGCAGCAGNNNNNN Phos
5’SOL- GN5-TAG CCACCGACAGGTTCAGAGTTCTACAGTAGCAGCAGGNNNNN Phos
5’SOL-lower-TAG Phos CTGCTGCTACTGTAGAACTCTGAACCTGTCGGTGG NH2

5’SOL- N6-**TGG** CCACCGACAGGTTCAGAGTTCTACAGTGGCAGCAGNNNNNN Phos
5’SOL- GN5-TGG CCACCGACAGGTTCAGAGTTCTACAGTGGCAGCAGGNNNNN Phos
5’SOL-lower-TGG Phos CTGCTGCCACTGTAGAACTCTGAACCTGTCGGTGG NH2

2nd SOL primer Bio CCACCGACAGGTTCAGAGTTCTACAG

3’ linker

3’ upper linker NNTCGTATGCCGTCTTCTGCTTG
3’ lower linker CAAGCAGAAGACGGCATACGA

PCR primers

PCR forward primer AATGATACGGCGACCACCGACAGGTTCAGAGTTC
PCR reverse primer CAAGCAGAAGACGGCATACGA

Sequencing primer

CGGCGACCACCGACAGGTTCAGAGTTCTACAG

3. CAGE BIOINFORMATICS AND QUALITY CONTROL

The reads were at first split according to their barcodes using a custom Perl script split_library.pl (available on request). Then, the quality and length of the reads was assessed using FastQC programme (Andrews, 2010) and summarized using MultiQC programme(Ewels et al., 2016). One barcode (CTT) was found to have a large number of short sequences that were attributed to contamination from an Illumina barcode. The reads were then trimmed from nested 5' and non-nested 3' adapters using Cutadapt (Martin, 2011). Single end reads were then aligned to hg38 (downloaded in September 2016 from UCSC: http://hgdownload.cse.ucsc.edu/goldenPath/hg38/bigZips/, release Dec 2013) with Burrows Wheeler Alignment (BWA) (Li and Durbin, 2009) using commands aln and samse. Between 3 million and 33 million reads (median 9 million) could be mapped. The expression levels (number of reads) were normalised with the R/Bioconductor package CAGEr (Haberle et al., 2015) using the getCTSS and normalizeTagCount commands with options "powerLaw" and an alpha value of 1.17, producing expression level estimates for each transcription start site (TSS) as TPM (tags per million). The normalised data were then formatted into OSCtable (details found at https://zenbu-wiki.gsc.riken.jp/zenbu/wiki/index.php/OSCtable), providing chromosome, start, end and strand coordinates. A header was included as specified in the Zenbu website and the file was uploaded into Zenbu (found at http://fantom.gsc.riken.jp/zenbu/) (Severin et al., 2014). Normalised promoter TPM values were taken from the Zenbu website.

TSS were grouped based on their distance apart using the distclu option in the CAGEr package (Haberle et al., 2015). The settings were as follows: the minimum TSS TPM value was 1, the distance between TSS was maximum of 20 bp and singletons (single TSS not neighbouring any other TSS) were not removed. To be able to compare the grouped TSS across different samples, the TSS range values needed to be aggregated. In this step, only the TSS groups with expression in at least one sample of higher than 5 TPM were kept, the others were filtered out. The maximum distance between TSS was kept at 100 bp. These commands created a single matrix file with cluster coordinates (start, end, strand) and normalised TPM values for the aggregated groups of TSS. All tags within an aggregated group of CAGE TSSs were allocated to the gene closest to the middle of the group region. These values were the expression level for that gene from the specific promoter represented by the aggregated group of CAGE tags. These aggregated groups of CAGE tags are also known as clusters of transcription start sites (CTSS) and this abbreviation is used in the text.

4. QRT-PCR VALIDATION OF CAGE GENE EXPRESSION LEVELS

Two sets of primers for two different housekeeping genes were used in this study. The first one was a published primer set for the human beta actin gene (*ACTN*) {Maess 2010}; the second for *GAPDH* was purchased from Qiagen (QuantiTect Primer Assay, QT0112646, Hilden, Germany). The rest of the primers were designed to span an intron, to have melting temperature (T_m) of 60°C and the cDNA product to be around 200bp using Primer3 programme (http://primer3.ut.ee/). Primer sequences are given in **Supplementary Table 2.** The ideal slope value from standard curves is -3.345 (when the primer efficiency is 2), but values from -3.0 to -3.5 were considered acceptable

Primers used for qRT-PCR validation of CAGE gene expression levels. The slope value for each primer set was calculated from the standard curves using Roche Light Cycler 480 software.

GENE TARGET	SEQUENCE 5' - 3'	SLOPE VALUE
**ACTB_F**	ATTGCCGACAGGATGCAGAA	-3.398
**ACTB_R**	GCTGATCCACATCTGCTGGAA	
**GAPDH**	Qiagen	-3.498
**MYB_EX5-6_F**	ACAGATGGGCAGAAATCGCA	-3.483
**MYB_EX5-6_R**	GCAGGGAGTTGAGCTGTAGG	
**CD14_EX2-3_F**	TAAACTGTCAGAGGCAGCCG	-3.345
**CD14_EX2-3_R**	TCGTCCAGCTCACAAGGTTC	

References for File S1

Andrews, S. (2010). *FastQC, a quality control tool for high throughput sequence data* [Online]. Babraham Bioinformatics. Available: http://www.bioinformatics.babraham.ac.uk/projects/fastqc [Accessed 2017].

Ewels, P., Magnusson, M., Lundin, S., and Kaller, M. (2016). MultiQC: summarize analysis results for multiple tools and samples in a single report. *Bioinformatics* 32**,** 3047-3048.

Haberle, V., Forrest, A.R., Hayashizaki, Y., Carninci, P., and Lenhard, B. (2015). CAGEr: precise TSS data retrieval and high-resolution promoterome mining for integrative analyses. *Nucleic Acids Res* 43**,** e51.

Li, H., and Durbin, R. (2009). Fast and accurate short read alignment with Burrows-Wheeler transform. *Bioinformatics* 25**,** 1754-1760.

Martin, M. (2011). Cutadapt removes adapter sequences from high-throughput sequencing reads. . *EMBnet* 17**,** 10-12.

Severin, J., Lizio, M., Harshbarger, J., Kawaji, H., Daub, C.O., Hayashizaki, Y., Bertin, N., and Forrest, A.R. (2014). Interactive visualization and analysis of large-scale sequencing datasets using ZENBU. *Nat Biotechnol* 32**,** 217-219.

Takahashi, H., Lassmann, T., Murata, M., and Carninci, P. (2012). 5' end-centered expression profiling using cap-analysis gene expression and next-generation sequencing. *Nat Protoc* 7**,** 542-561.
